# Factor Analysis of MYB Gene Expression and Flavonoid Affecting Petal Color in Three Crabapple Cultivars

**DOI:** 10.3389/fpls.2017.00137

**Published:** 2017-02-07

**Authors:** Jie Zhang, Yingying Liu, YuFen Bu, Xi Zhang, Yuncong Yao

**Affiliations:** ^1^Department of Plant Science and Technology, Beijing University of AgricultureBeijing, China; ^2^Key Laboratory of New Technology in Agricultural Application of Beijing, Beijing University of AgricultureBeijing, China; ^3^Beijing Collaborative Innovation Center for Eco-Environmental Improvement with Forestry and Fruit TreesBeijing, China

**Keywords:** *Malus* crabapple, flavonoids, petal pigmentation, factor analysis, gene expression pattern

## Abstract

Flavonoid biosynthesis has received much attention concerning the structural genes and expression of the associated transcription factors (TFs). In the present study, we examined the gene expression patterns for petals of three colors using a statistical method. Factor analysis was successfully used to examine the expression patterns most present during regulation. The first expression patterns in the white and red petals were clearly demonstrated and have revealed different mechanisms of producing the proper components, whereas that in the pink petals was more complex, requiring factor analysis to supplement the other results. Combining the results of the correlation analysis between TFs and structural genes, the effects of each TF on the main expression pattern in each cultivar were determined. Moreover, *McMYB10* was implicated in the regulation of the gene expression pattern in red petals, and *McMYB5* was implicated in the maintenance of the balance of the pigment components and proanthocyanin (PA) production in cooperation with *McMYB4* to generate pigmentation in the pink petals.

## Introduction

Crabapples, belonging to the *Malus* genus, are excellent ornamental and economic germplasm resources throughout the world, with diverse color variations in the fruits, flowers and leaves under the direct influence of flavonoids (Liu et al., 2011; Tan et al., 2013). The pigmentation of flowers is important for their ornamental value, with a vital role in sightseeing. Flower pigmentation is determined by the accumulation of anthocyanins (i.e., cyanidin-3-O-glucoside, delphinidin-3-O-glucoside, and pelargonidin-3-O-glucoside; Schwinn et al., 2006), associated with flower development and color change. Moreover, much is known about the functions of the precursors and residues included in the frontal biosynthesis pathway for UV-defense and chemical information exchange (Winkel-Shirley, 2001), antioxidant activity and protection against coronary heart disease, certain cancers, and senile diseases under the effect of flavonols, which are also synthesized through the flavonoid biosynthesis pathway (Hellens et al., 2010).

Derived from Phe (Phenylalanine) and malonyl-coenzyme A (CoA; via the fatty acid pathway), the flavonoid pathway includes six major subgroups of compounds: chalcones, flavones, flavonols, flavandiols, anthocyanins, and condensed tannins (or proanthocyanidins; Winkel-Shirley, 2001; Shelton et al., 2012). The initial step of flavonoid biosynthesis involves the synthesis of the common precursor p-coumaric acid by phenylalanine ammonialyase (PAL); subsequently, the chalcone naringenin is synthesized by chalcone synthase (CHS), and the branches from the forks at the beginning of the pathway are responsible for the synthesis of lignins or coumarins (Schenke et al., 2011; Shen et al., 2012). Beneath the function of PAL, cinnamate 4-hydroxylase (C4H), and 4-coumaroyl coenzyme A ligase (4CL) (Vogt, 2010), the biosynthesis procedures are known as the general phenylpropanoid pathway (GPP), followed by CHS, leading to the formation of pigmentation components, such as isoflavonoids, anthocyanins, 3-deoxyanthocyanidins and tannins. Influenced by dihydroflavonol 4-reductase (DFR) and anthocyanidin synthase (ANS) (Hancock et al., 2012), the primary color components in crabapples include pelargonidin-3-O-glucoside and cyanidin-3-O-glucoside (Liu et al., 2011), with naringenin at the fork before the two branches, as shown in **Figure 3**. In addition, the pathway for the synthesis of proanthocyanins (PAs) has been intensively explored in *Arabidopsis thaliana* and many other plants (Abeynayake et al., 2012). The significant accumulation of PAs occurs in light-colored petals, peels, flesh and the seed coat through the spatial and temporal expression of genes, and it maintains a relatively independent balance of expressed/repressed feedback mechanisms (Henry-Kirk et al., 2012). The PA branch stretches from leucoanthocyanidins and anthocyanidins to form catechins and epicatechins, respectively.

Previous studies have also shown that many transcription factors (TFs), such as *PtMYB14* in *P. taeda, CmMYB1* in chrysanthemum, and *MdMYB10* and *Md110a* in apple, are responsible for the flavonoid response (Espley et al., 2007; Lin-Wang et al., 2010; Chagné et al., 2013; Zhu et al., 2013). Additional studies have also demonstrated that one TF might affect several, even as many as 12, structural genes (Shelton et al., 2012). In contrast, the regulation of one structural gene might be induced through several TFs (Ravaglia et al., 2013). More previous studies have focused on the cooperation of two or more TFs to maintain the balance of the flavonoid pathway or the implicated feedback patterns (Schenke et al., 2011; Fornalé et al., 2013; Yuan et al., 2013). The gene *AtBAN* in *Arabidopsis* plays an important role in the PA pathway to promote or suppress the regulation of PA synthesis through R2R3-MYB TFs (Espley et al., 2007; Thevenin et al., 2012), which also involves a feedback pattern between LAR/ANR and the components of the PA synthesis pathway (Henry-Kirk et al., 2012).

In general, the results of the empirical analysis of the gene expression levels are used to determine the relationships among genes. The variation trend of genes gives researchers a general idea of the biosynthetic activity, without statistical accordance, or inner correlation beneath the values (Matoušek et al., 2012). Indeed, gene expression patterns are frequently explained as a common factor that encompasses all the involved genes and represents a portion of each of the genes implicated in these patterns (Pournara and Wernisch, 2006; Cheng et al., 2007; Bagyamani and Thangavel, 2010). Thus, we used an adequate statistical method, factor analysis, which is typically used in the humanities, sociology, and economics to extract common factors among many values (Holtzer et al., 2006; An et al., 2011; Park, 2014). When examining problems in gene expression, the expression patterns are determined by factor analysis extracting the relevant genes as common factors. However, statistical methods are seldom considered for the analysis of molecular problems. Considering differences in the genetic backgrounds among the three cultivars and the inconformity among gene expression levels, a statistical analysis is a reasonable method for confirming the basic values.

The purpose of the present study was to integrate and compare the color formation patterns of the gene expression levels in red, pink, and white flowers. We measured the correlation among the structural genes and some R2R3-MYBs by quantitative real-time PCR (qRT-PCR) at five development stages in flowers of three colors. The gene expression levels were calculated through factor analysis to determine the expression patterns in each cultivar. The results of former studies and the expression analysis and cooperation between the structural genes and TFs observed in the present study suggest that *McMYB10* is involved in the regulation of anthocyanin production in red petals (Jiang et al., 2013), whereas *McMYB5* and *McMYB4* cooperate to maintain the balance between the pigment components and PA production in pink petals.

## Materials and methods

### Plant material

*Malus* cv. “Royalty,” *Malus* cv. “Radiant,” and *Malus* cv. “Flame” flowers were collected at different developmental stages (1, 6 days before full bloom; 2, 3 days before full bloom; 3, 1 day before full bloom; 4, full bloom; and 5, bloomed). All the petals of 10 flowers were collected and measured in further study. Each study was performed in at least three biological replications. All samples were frozen in liquid nitrogen and stored at −80°C.

### High-performance liquid chromatography (HPLC) analysis of the anthocyanin and flavonoid content

Anthocyanins and flavonoids were extracted using a methanol:water:formic acid:trifluoroacetic acid solution (70:27:2:1, v/v). The supernatants were filtered through a 0.22 μm Millipore™ filter (Billerica, MA, USA) prior to use. The anthocyanins in the samples were analyzed using an HPLC1100-DAD system (Agilent Technologies, Waldbronn, Germany). Detection was performed at 520 nm for anthocyanins and 350 nm for flavonoids. A solid-phase C18 Supelclean ENVI-18 extraction cartridge (500 mg, 3 ml) was used for separation at 30°C, and elution was conducted with a mobile phase comprising solvent A, trifluoroacetic acid:formic acid:water (0.1:2:97.9) and solvent B, trifluoroacetic acid:formic acid:acetonitrile:water (0.1:2:35:62.9) at a flow rate of 0.8 ml min^−1^. The elution program was performed according to Wu and Prior (2005), with several modifications. Briefly, solvent B started at 30% and increased linearly stepwise to 35% at 5 min, 40% at 10 min, 50% at 30 min, 55% at 50 min, 60% at 70 min, and 30% at 80 min. The standards used for the HPLC analysis are shown in Figure S1.

### qRT-PCR analysis

Total RNA from flower tissues was extracted using an EASYspin Plus Plant RNA Kit (Aidlab, Beijing, China) according to the manufacturer's instructions. DNase I (TaKaRa, Japan) was added to remove genomic DNA, and the samples were subjected to cDNA synthesis using the Access RT-PCR System (Promega, USA) according to the manufacturer's instructions. RNA samples (1 μg) were reverse transcribed into complementary DNA (cDNA) using an oligo (dT) 18 primer and M-MLV reverse transcriptase (TaKaRa) following the manufacturer's protocol. qRT-PCR was carried out using SYBR® Premix Ex Taq™ II(Perfect Real Time) (TaKaRa) on the CFX96™ Real Time System (Bio-Rad). The differences in gene expression were calculated using the 2^∧(−ΔΔCt)^ analysis method, and the transcription levels were determined by relative quantification using the *Malus* 18S ribosomal RNA gene as the reference gene.

The qPCR analysis was conducted in a total volume of 20 μl containing 9 μl of 2 × SYBR Green qPCR Mix (Takara, Japan), 0.1 μM specific primers (each), and 100 ng of template cDNA. The reaction mixtures were heated to 95°C for 30 s, followed by 39 cycles at 95°C for 10 s, 59°C for 15 s, and 72°C for 30 s. The sequence and the information of the primers used in the qPCR are presented in Table S1 and Figure S2.

### Statistical analysis

HPLC and qRT-PCR assays were analyzed at least in biological triplicate. All data were analyzed using one-way ANOVA, followed by Duncan's SSR test (shortest significant ranges) to compare differences among the experimental sites at *P* < 0.05 [Microsoft Excel 2010, Statistical Product and Service Solutions (SPSS v19.0) and Origin v7.05]. qPCR data of the structural genes were analyzed using factor analysis to compare the differences between principal components with SPSS v19.0.

### Accession numbers

Accession numbers of the genes used in this article are shown in Table S1.

## Results

### The pigment concentration affects the flower phenotype

**Figure 1:**
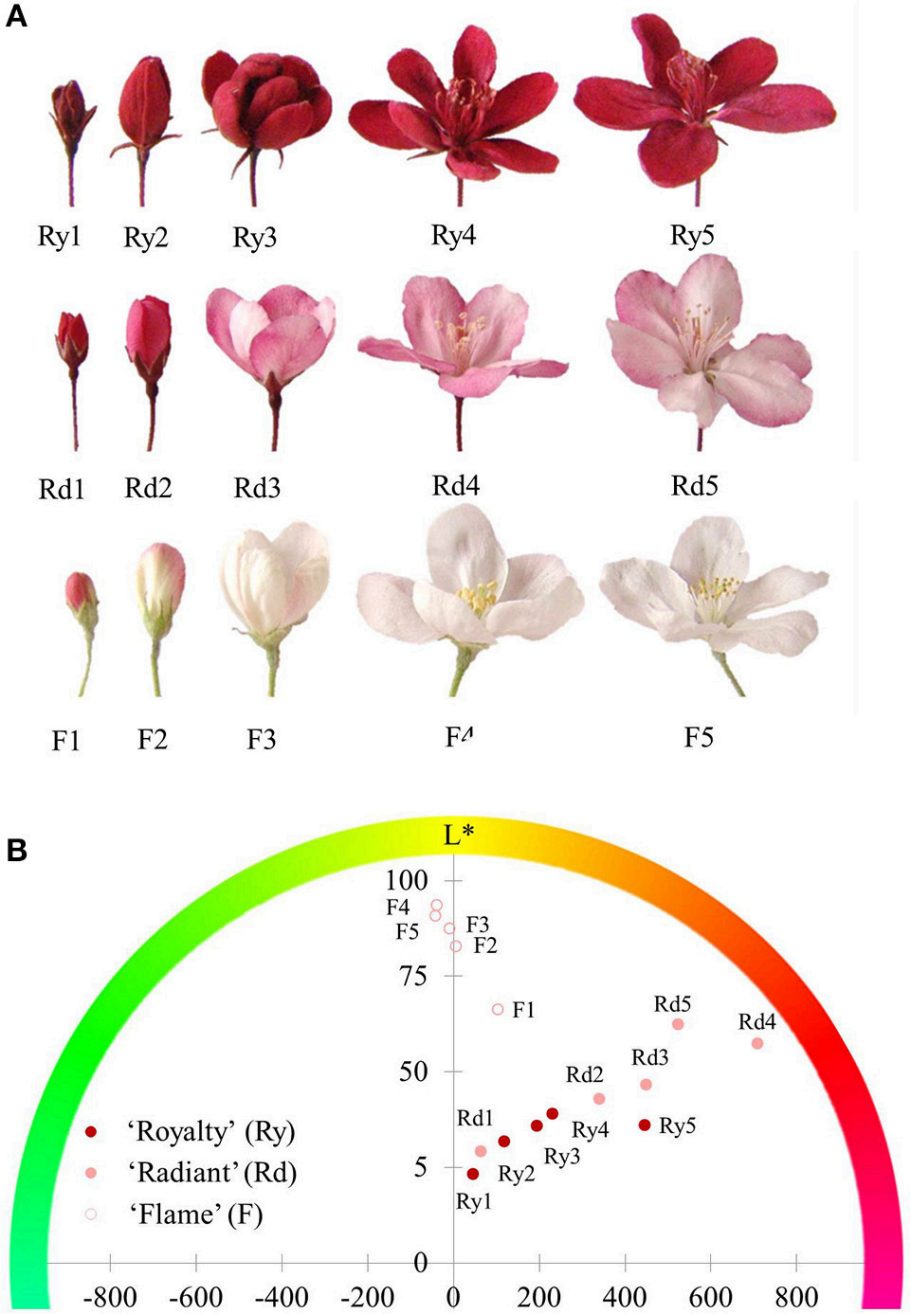
**Flower phenotypes of three crabapple cultivars during flower development. (A)** Flower blossom status. The images shown from left to right display the five development stages: 1-small bud stage; 2-large bud stage; 3-initial bloom stage; 4-full bloom stage; and 5-bloomed stage. **(B)** Hue value in the coloring coordinate. Ry1-Ry5, Rd1-Rd5, and F1-F5 represent the five flower development stages of “Royalty,” “Radiant,” and “Flame,” respectively. Differences in the hue angle and lightness (L^*^) among the three cultivars. The included angles between the dots and *x*-axis refer to the color on the outlying hue circle. The height of the *y*-axis indicates the degree of lightness (L^*^).

The different color phenotypes of crabapple cultivars are shown in Figure 1A, in which “Royalty” is a typical red flower cultivar, “Radiant” is a pink flower cultivar, and “Flame” is a white flower cultivar. The flower color of “Radiant” and “Royalty” flower blossoms faded from the small bud to the bloomed stage, whereas that of “Royalty” showed no changes. These effects might be associated with pigment accumulation.

To investigate differences in the three flower color phenotypes, we measured the hue angle (Ha) and lightness (L^*^) of the flowers during development stages using a digital color-difference meter. The hue angle is a measurement of color concentration, which facilitates the analysis of color as a percentage of the three primary colors. To some extent, lightness reveals the density of the pigments located in the same material area. The position of the dots shown in Figure 1B indicated that both the red flowers of “Royalty” and the pink flowers of “Radiant” varieties shared the same red coloring type (Ha) but displayed differences in the lightness level (L^*^), with pink flowers showing an obvious advantage over red flowers in the L^*^ levels at every development stage. The L^*^ level of red flowers did not increase during later stages of development, whereas that of pink flowers continuously increased. In addition, the tint area of the white flowers increased during development, and the L^*^ level was consistently higher than that of pigmented flowers. These results indicated that the petals of red and pink flowers had a high level of pigment accumulation associated with red color.

**Figure 2:**
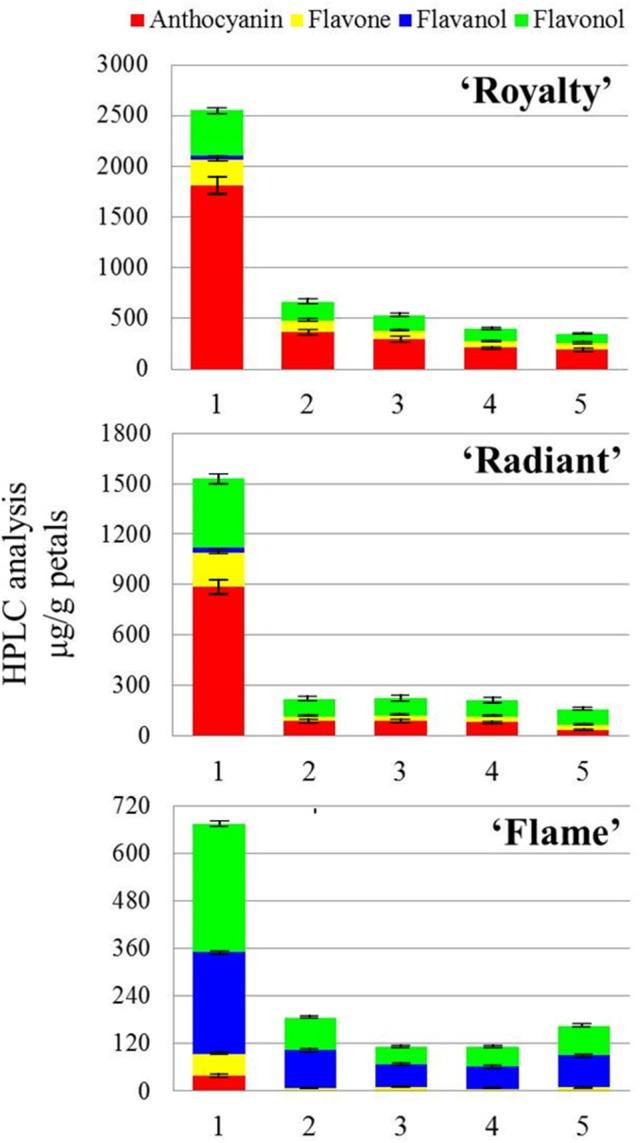
**HPLC analysis of the changes in the flavonoid expression in the flower petals of three cultivars during flower development**. HPLC analysis of three crabapple cultivars in the present study. The compositions identified in the HPLC analysis are divided into four types: anthocyanin, flavone, flavanol, and flavonol, shown in four colors in the legends. The numbers 1–5 refer to five flower development stages.

The HPLC analysis of flower petal coloring among the three cultivars based on flavonoid biosynthesis revealed six important pigments, divided into four groups according to chemical structure: anthocyanins, including cyanidin-3-O-glucoside and pelargonidin-3-O-glucoside, which is primarily responsible for red color pigmentation; flavanols, such as catechin, which are responsible for PA synthesis; flavones, including apigenin; and flavonols, including quercetin, rutin, and aglucon (Figure 2).

As shown in Figure 2 and Table 1, anthocyanins were the predominant pigment detected in red petals, whereas in white petals, low concentrations of this pigment were observed during the young bud development stage. Except for concentration, the proportion of anthocyanins detected in white petals was significantly lower than that in red petals. A comparison of the HPLC results between pink and red petals revealed that the proportion of anthocyanin was lower in pink (56.93% with stage 1, 28.31% with stage 2, 29.43% with stage 3, 32.17% with stage 4, 16.01% with stage 5) petals than in red (70.35% with stage 1, 55.65% with stage 2, 52.84% with stage 3, 54.11% with stage 4, 52.07% with stage 5) petals. Indeed, the concentration of anthocyanin in pink petals was nearly half of that in the red petals. Interestingly, compared with stage 1 flowers in all three, the concentrations of anthocyanin with stage 2–5 were reduced (Table 1).

**Table 1:** **Contents of the flavonoids in the three cultivars**.

**Cultivars**	**Contents of flavonoids (μg/g)**				
		**Anthocyanins**	**Flavones**	**Flavonols**	**Flavonols**
Royalty	Ry1	1805.65 ± 37.47	252.20 ± 4.39	477.12 ± 11.21	31.79 ± 1.54
	Ry2	380.40 ± 8.76	104.87 ± 2.13	198.31 ± 3.46	N/A
	Ry3	276.91 ± 10.28	82.61 ± 1.06	164.58 ± 3.45	N/A
	Ry4	226.65 ± 6.35	61.76 ± 1.28	130.49 ± 2.30	N/A
	Ry5	184.99 ± 7.23	60.57 ± 1.28	109.67 ± 2.80	N/A
Radiant	Rd1	872.80 ± 21.79	206.17 ± 6.54	432.16 ± 12.16	22.06 ± 1.63
	Rd2	54.03 ± 3.21	29.76 ± 1.13	107.08 ± 2.26	N/A
	Rd3	58.86 ± 2.68	34.98 ± 1.75	106.00 ± 2.47	N/A
	Rd4	59.32 ± 3.68	25.60 ± 2.01	99.51 ± 1.18	N/A
	Rd5	23.20 ± 1.13	33.40 ± 2.35	88.23 ± 1.31	N/A
Flame	F1	33.66 ± 2.25	24.56 ± 2.24	343.73 ± 10.55	259.89 ± 5.13
	F2	N/A	8.57 ± 0.53	79.97 ± 2.82	95.32 ± 2.17
	F3	N/A	8.22 ± 0.77	44.52 ± 1.54	58.15 ± 2.43
	F4	N/A	5.81 ± 0.64	47.05 ± 1.18	58.84 ± 2.76
	F5	N/A	8.64 ± 0.81	70.39 ± 1.63	75.01 ± 4.43

*The error bars represent the mean ± SD of three biological replicates*.

### Factor analysis of the expression the structural genes involved in the flavonoid synthesis pathway

**Figure 3:**
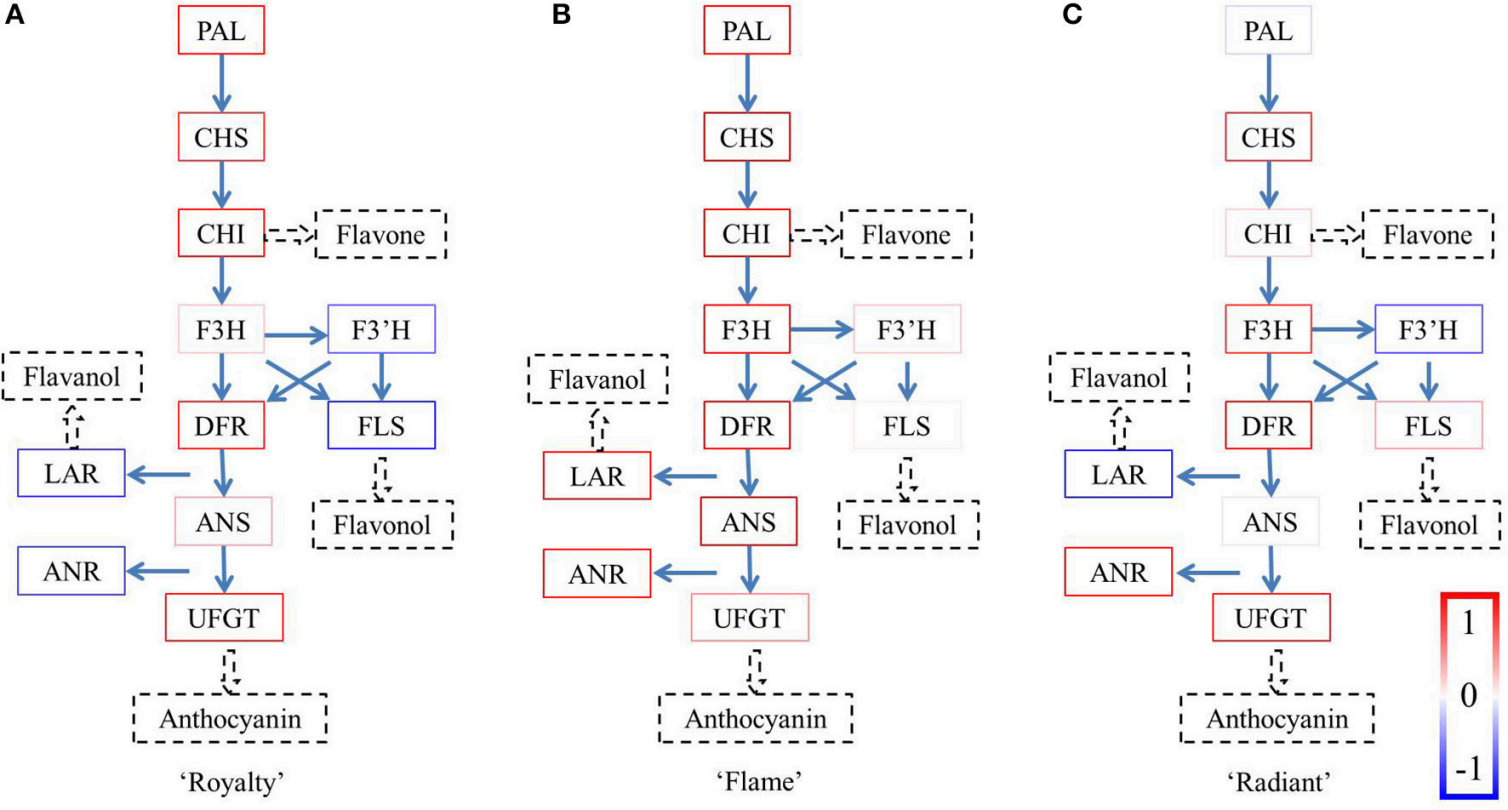
**Factor analysis of the expression levels of the structural genes**. The results calculated between −1 and 1 are expressed as boxes in the colors shown on the bottom right. The boxes for each gene were integrated into one pathway for each of the three cultivars. **(A)** Royalty, **(B)** Flame, and **(C)** Radiant.

The factor analysis was based on the expression of the structural genes involved in the flavonoid synthesis pathway. qRT-PCR revealed variations in the transcriptional expression of structural genes during different flower development periods, and these data were further used to identify common factors using SPSS software. The composition of the common factors indicates the relationship among the different structural genes at the gene expression level and provides evidence of the gene expression patterns (Figure 3, Figure S3). Considering the anthocyanin is responsible for red color pigmentation, these variations in the proportion of anthocyanin were consistent with the coloration changes of the three cultivars with the development stages.

**Figure 4:**
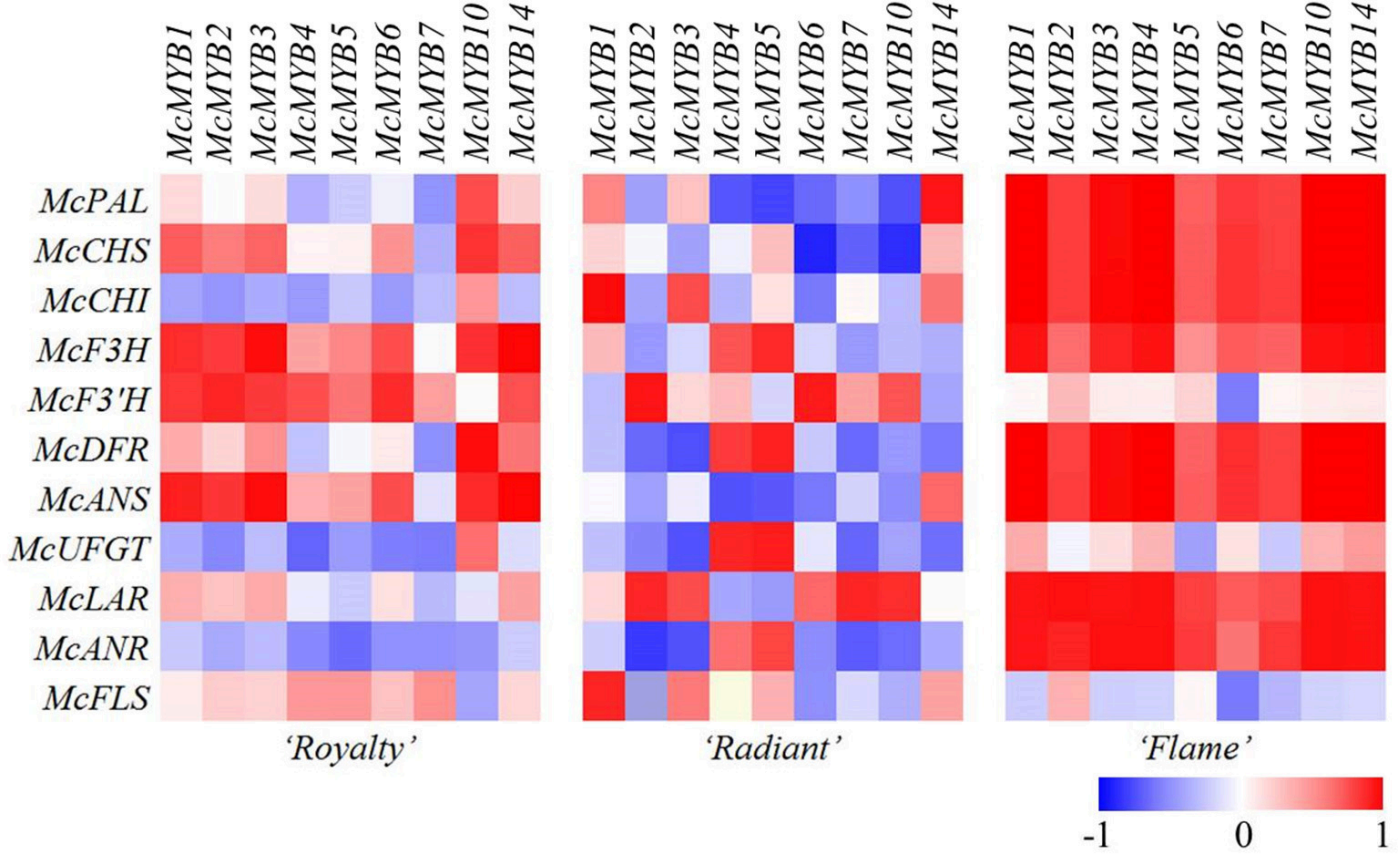
**Heat map showing the varying intensities of the three independent cultivars, maximum scaled to one transcription factor for each expression**. Relative quantification of the expression of the annotated genes using qRT-PCR. 1: *McMYB1*, 2: *McMYB2*, 3: *McMYB3*, 4: *McMYB4*, 5: *McMYB5*, 6: *McMYB6*, 7: *McMYB7*, 8: *McMYB10*, 9: *McMYB14*.

There were two factors with initial eigenvalues >1 in “Royalty” and “Flame,” respectively, whereas in “Radiant,” there were three factors with eigenvalues >1 (Tables S2–S7). In each factor, the structural genes own their own component varying between –1 and 1. The colors were defined according to the values for the structural components, varying from blue to white when the components were negative and from red to white when the components were positive (Figure 4). The colors of the components in the first factor reflected the genes involved in the flavonoid biosynthesis pathway. Only the factors with the highest initial eigenvalues in each cultivar showed clear expression patterns. In the other factors, the majority of the components reflected in the flavonoid biosynthesis pathway were shown in light colors, and even the structural genes with highest components did not show a clear expression pattern. Thus, considering the biological significance of these data, the structural gene expression patterns were only observed for the factors with the highest initial eigenvalues.

In “Royalty” (Figure 3A), the status of *McPAL, McCHS, McCHI McDFR*, and *McUFGT*, in bright red colors, suggested that these components form an entire branch of the anthocyanin synthesis pathway, except for *McF3H* or *McANS* in weak red colors, which is a lower component. However, the genes in the flavanol and flavonol branches occupied negative components, indicated as blue boxes, suggesting that these components have little effect on the synthesis of flavanols and flavonols.

The schematic diagram of the flavonoid biosynthesis pathway in “Flame” (Figure 3B) reveals that the pathway from *McPAL* to *McANS* is complete with respect to the bright red boxes, indicating the intensity effect. However, the high levels of *McLAR* and *McANR* implicated the activity of the PA branch, compared with the lower component *McUFGT*, indicated in light red. Moreover, the genes component in the flavonol branch occupied lower components, indicated as light red boxes.

Compared with “Royalty,” the gene expression pattern for the pigment biosynthesis pathway in “Radiant” (Figure 3C) was not complete, ending with the high component *McUFGT*, which was supposed to ensure sufficient synthesis of anthocyanins. Notably, compared with “Flame,” only half of the branch for PA synthesis in this variant was completed. The component of *McANR* was significantly positive, whereas that of *McLAR* was significantly negative, consistent with the idea of feedback regulation.

### Correlation of structural genes and MYB TFs

We constructed a heat map based on the results of the correlation analysis (Figure 4). The white petals of “Flame” showed an obvious change in the gene expression pattern, whereas the red petals of “Royalty” showed a more varied expression pattern, and the changes in the expression patterns of the pink petals of “Radiant” showed the most complex expression, suggesting a multi-aspect cause of middle petal color formation at the gene expression level.

The expression patterns of the structural genes during flower development in “Flame” showed a remarkable correlation (indicated as bright red boxes) compared with that in the other two species. The MYBs were indicated as positive regulators in high or remarkable correlation with the expression of the structural genes, which are barely expressed in plants, suggesting an alternative possibility. However, the *McUFGT* status shows a low correlation with the structural genes in the white petals, suggesting that *McUFGT* acts differently under the influence of other MYBs.

The expression of MYBs was significantly correlated with that of the structural genes with high components in the first factor or the gene expression pattern in “Flame.” However, the MYBs also showed grids in light colors, revealing a low correlation with *McF3*′*H, McFLS*, and *McUFGT* (Figure 4).

In “Royalty,” the MYBs showed a low correlation with the genes involved in the pathways for the synthesis of PA and flavonol. However, *McMYB1, McMYB2, McMYB3, McMYB10*, and *McMYB14* showed a more significant correlation with the genes involved in the anthocyanin synthesis pathway from *McPAL* to *McUFGT*. *McMYB10* showed a more significant correlation with the majority of the genes with high components in the first factor, particularly *McCHS* and *McDFR*, consistent with the experimental results. When observing the crosswise orientation to *McDFR, McCHS*, and *McUFGT*, only *McMYB10* revealed a significant correlation (Figure 4).

In “Radiant,” the percentage of negative correlation was much higher than that observed in the other two cultivars. The portrait orientation showed an obvious correlation of a few structural genes with MYB. Similar to the status of “Royalty,” *McMYB5* showed a significant correlation with the structural genes in high components in the first factor of “Radiant.”

## Discussion

### The results of the factor analysis indicated the gene expression patterns of the anthocyanin synthesis pathway

Factor analysis has been commonly used for decades to resolve problems in the humanities, sociology, and economics (Holtzer et al., 2006; An et al., 2011; Park, 2014). Using principal component analysis as a reference, factor analysis is a statistical method used to explore the dependence of original variables on the inner correlation matrix and present the dependence of complex variables, such as common factors, on linear transformation (Suhr, 2006; Eren et al., 2012).

Factor analysis has a great potential for developing and establishing the genotype and phenotype model. The Factor Analysis Model (FAM) framework plays an important role in incorporating important gene ontology information and solving the problem in using statistical classification techniques for functional genomics (Kustra et al., 2006). Factor analysis can also be applied to other related fields, such as the analysis of gene and protein expression data (Tan et al., 2009; Blum et al., 2010). In a previous study, the expression of *AtACS2, AtACS7, AtACS8, AtACS11, AtACO*, and *AtACO2*, which are pivotal enzymes in the ethylene biosynthetic pathway, were dramatically upregulated and related in response to excess Fe (Li et al., 2015). An early study with crabapple (*M. xiaojinensis*) suggested that the transcription level of the *MxFRO*_2_ gene in *M. xiaojinensis* roots is closely associated with the chlorosis phenotype (Wu et al., 2012). These results showed that the gene expression may represent the phenotype in plants. In our study, two common factors were extracted from “Royalty” and “Flame,” and three common factors were extracted from “Radiant.” Considering the statistical significance of these data, the factors with total initial eigenvalues >1 are typically considered referable. Considering the biological significance, these factors were analyzed using an empirical analysis method. The structural genes identified in the present study have been implicated in the regulation of flavonoid synthesis (Lin-Wang et al., 2010, 2011; Telias et al., 2011; Sobel and Streisfeld, 2013). The structural gene components in the same common factor or gene expression pattern are reflected in a series of colors.

Defined as red boxes, the components >0 are considered important information when analyzing the major gene expression patterns in each cultivar (Figure 3). The genes indicated in bright red jointly form a gene expression pattern. The genes shown in blue have negative components, and those displayed in light red may not be associated with the gene expression pattern but might be important for regulation of synthesis. Thus, we primarily focused on the genes shown in bright red boxes. Therefore, only the patterns of the first factors containing the majority of genes in bright red boxes were used to determine the respective gene expression pattern, whereas the genes in other factors showed very few clear expression patterns or were not displayed as bright red boxes.

The gene expression patterns of the first factors identified in “Flame” were implicated in the flavanol synthesis pathway as genes in bright red boxes. *McF3*′*H* and *McFLS* in the branch for flavonol synthesis and *McUFGT* in the branch for anthocyanin synthesis are shown in light red boxes. Together with the results of the HPLC analysis, the concentration of anthocyanins and flavanols, the main pigment components, matches the first pattern in “Flame.” In white flowers, the flavanol branch intensely competes with the anthocyanin branch and shares a slight effect with *McUFGT*, as indicated in the light red box. Briefly, the gene expression pattern of anthocyanin synthesis in “Flame” clearly shows that the flavanol branch primarily contains the genes involved in the synthesis of white pigment components (Hellens et al., 2010; Debes et al., 2011; Fraser et al., 2013). Meanwhile, these results are consisted with our previous study of “Flame” petals (Jiang et al., 2013).

The results of the factor analysis and HPLC in “Royalty” showed that the anthocyanin branch exceeds the other branches containing genes in bright red boxes from *McPAL* to *McANS*, except for *McF3H* and *McANS*. In pure red flowers, it is likely that *McF3H*, indicated in a light red box, might have a restrictive effect at the fork of the flavonol branch and *McANS* might have a restrictive effect at the fork of the anthocyanin branch, with *McDFR*, indicated in a bright red box, situated between these two pathways to ensure the adequate production of intermediate products. In crabapple, the relative activities of McDFR and McFLS are important determinants of the red color of crabapple leaves, via the regulation of the metabolic fate of substrates that these enzymes have in common (Tian et al., 2015); therefore, the present study further confirmed this regulation mechanism in crabapple flowers.

The results of the factor analysis for “Radiant” were not as clear as those obtained for the white or red flowers. The HPLC analysis showed that the concentration of the pigment components in the pink and red flowers was similar, with anthocyanin as the predominant pigment. The difference in anthocyanin concentration in “Royalty” was higher than that in “Radiant.” Compared with the expression pattern observed in “Royalty,” the early biosynthesis genes *McPAL* and *McCHI*, displayed in light red boxes, might play restrictive roles, controlling the total concentration of the pigment products. The late biosynthesis gene *McUFGT*, indicated in a bright red box, contributes to anthocyanin synthesis for pink flower pigmentation. Briefly, the gene expression pattern of the anthocyanin synthesis pathway in “Radiant” is complex but produces anthocyanin with a limit on the total concentration of this pigment produced in earlier steps of the flavonoid pathway.

The flavonol concentration was similar in the three cultivars (Figure 2), which is not well-reflected in the first common factor. However, the components of *McF3*′*H* and *McFLS* indicated that flavonol is a second factor, showing an incomplete pattern. We found that *McFLS* expression and flavonol accumulation were positively correlated in the flowers of these three cultivars and that the *McFLS* gene played an important role in flavonol biosynthesis. These results were similar to those regarding the function of FLS in petunia petals and crabapple leaves (Davies et al., 2002; Tian et al., 2015).

### *McMYB10* and *McMYB5* are involved in the regulation of the gene expression patterns in “royalty” and “radiant,” respectively

Previous studies showed that some R2R3-MYB TFs not only significantly affect several genes in the same metabolism process but also simultaneously regulate these genes (Fornalé et al., 2013; Rahim et al., 2014), which likely also affects many other metabolic processes (Gonzalez et al., 2009; Bedon et al., 2010; Yuan et al., 2013; Liu et al., 2014). The correlation of the expression levels of TFs revealed that the expression of MYB affects more than one structural gene. Furthermore, the factor analysis indicated that the TFs are highly correlated with the structural genes indicated in bright red boxes in the first factor associated with the first gene expression pattern.

The factor analysis of the TFs in “Royalty” showed that *McMYB10* is highly correlated with all of the structural genes indicated in bright boxes in the first expression pattern, consistent with previous studies on anthocyanin biosynthesis, showing that *MYB10* is involved in the regulation of anthocyanin biosynthesis in apples, strawberries and peaches (Espley et al., 2007; Telias et al., 2011; Fraser et al., 2013). The inhibiting TF *MYB7* shows a low correlation with the first expression pattern, confirmed as a repressor of *DFR, UFGT*, and *CHI* in several different tissues. Moreover, *McMYB1, McMYB2, McMYB3*, and *McMYB14* are highly correlated with approximately half of the structural genes in the anthocyanin pathway. *McMYB14*, which plays a role in the defense response as a flavonoid regulator, is also involved in other metabolic processes (Höll et al., 2013; Fang et al., 2014; Duan et al., 2016). Considering the function of *McMYB1, McMYB2*, and *McMYB3* in metabolism, more or less associated with the defense response (Wei et al., 2011; Baek et al., 2012; Liu et al., 2014), it is inferred that the expression levels of these genes might vary with metabolism, synchronizing with the flavonoid pathway involved in the defense response.

Only *McMYB4* and *McMYB5* shared a high correlation with the first expression pattern in “Radiant.” Studies have shown that some R2R3-MYB TFs cooperate to regulate flavonoid biosynthesis and maintain the pathway balance (Fornalé et al., 2013; Yuan et al., 2013; Liu et al., 2014). *MYB5* is closely associated with PA biosynthesis, tannin production and even testa development (Cavallini et al., 2013) and has been indicated as an important regulator of the reduction of pigment components and the accumulation of PA (Liu et al., 2014). *MYB4* likely suppresses *MYB7* to remove the inhibition of DFR and UFGT to maintain the production of pigment components (Fornalé et al., 2013). Thus, based on the evidence discussed above, *McMYB4* and *McMYB5* are speculated to cooperate and maintain the balance in flavonoid biosynthesis in pink petals.

In summary, the biosynthetic regulation mechanism of flavonoids in the flowers of different crabapple cultivars are complex, and several MYB TFs are involved in this biosynthesis process. Furthermore, the expression of flavonoid biosynthetic genes may be coordinately regulated by several MYB TFs in crabapple flowers.

We hope the work described in this report will provide a research basis to build the genotype and phenotype model and that our results will improve the flavonoid pathway network and provide new perspectives for ornamental fruit tree breeding.

## Author contributions

YL, JZ, and YB designed research; YL and YB performed research; XZ and JZ analyzed data; JZ and YY wrote the paper.

### Conflict of interest statement

The authors declare that the research was conducted in the absence of any commercial or financial relationships that could be construed as a potential conflict of interest.
