# Supplementary figures and images for: Factor Analysis of MYB Gene Expression and Flavonoid Affecting Petal Color in Three Crabapple Cultivars
Source: Front Plant Sci. 2017 Feb 7;8:137. doi: 10.3389/fpls.2017.00137 (PMC5293739; doi:10.3389/fpls.2017.00137)

Table S1. List of the oligonucleotide primers for qRT-PCR.


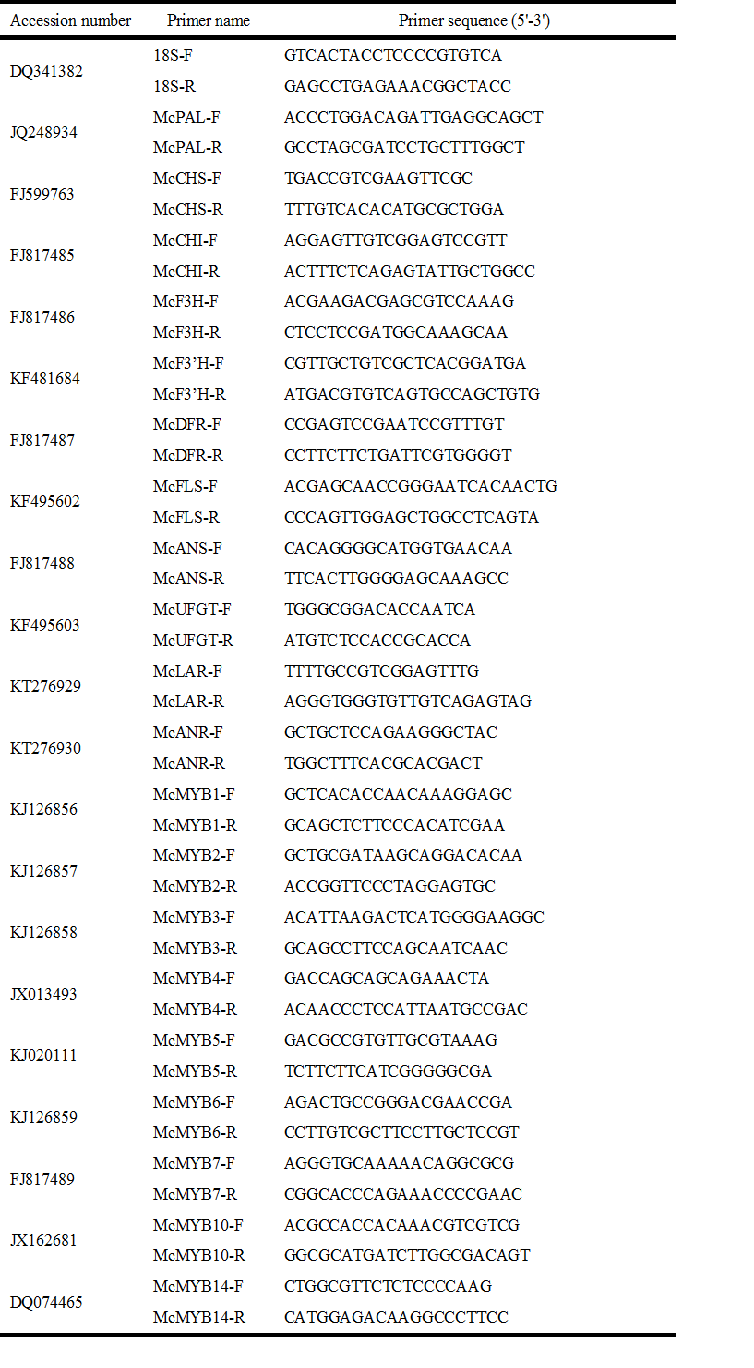

Supplement: Supplementary file 1 [file Table1.DOCX]

Table S2. Total variance explained in ‘Royalty’.


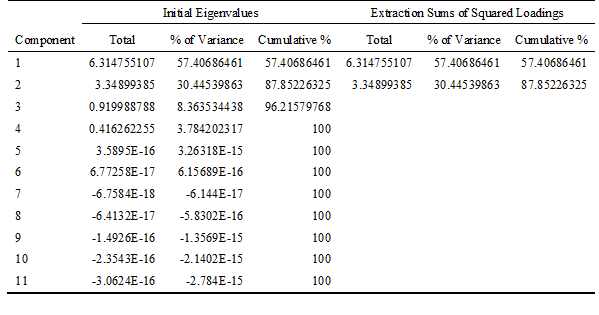

Supplement: Supplementary file 2 [file Table2.DOCX]

Table S3. Component matrix of two factors that are greater than 1 in ‘Royalty’.


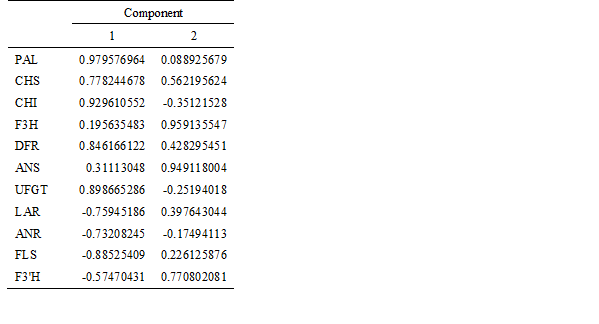

Supplement: Supplementary file 3 [file Table3.DOCX]

Table S4. Total variance explained in ‘Radiant’.


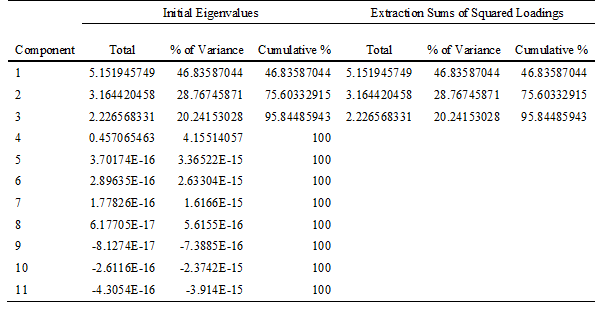

Supplement: Supplementary file 4 [file Table4.DOCX]

Table S5. Component matrix of three factors that are greater than 1 in ‘Radiant’.


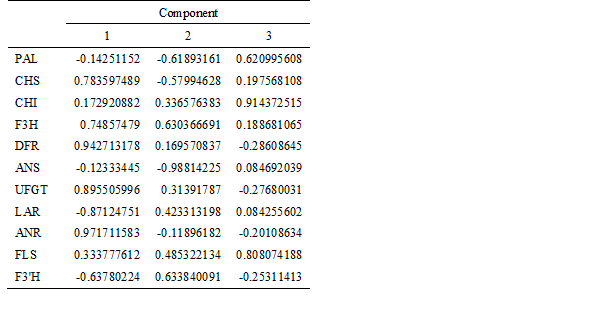

Supplement: Supplementary file 5 [file Table5.DOCX]

Table S6. Total variance explained in ‘Flame’.


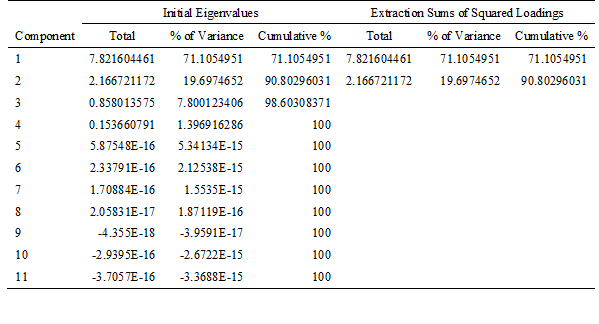

Supplement: Supplementary file 6 [file Table6.DOCX]

Table S7. Component matrix of two factors that are greater than 1 in ‘Flame’.


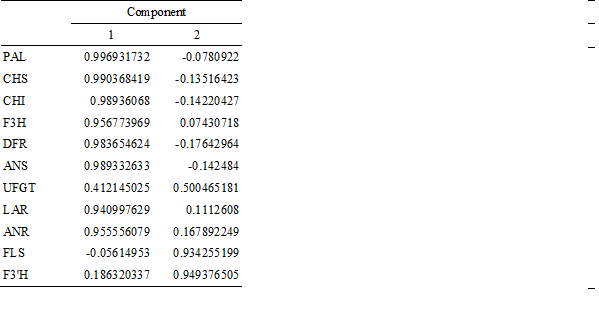

Supplement: Supplementary file 7 [file Table7.DOCX]
