# Supplementary material for: Factor Analysis of MYB Gene Expression and Flavonoid Affecting Petal Color in Three Crabapple Cultivars
Source: Front Plant Sci. 2017 Feb 7;8:137. doi: 10.3389/fpls.2017.00137 (PMC5293739; doi:10.3389/fpls.2017.00137)
Supplement: Supplementary file 8 [file Image1.PDF]

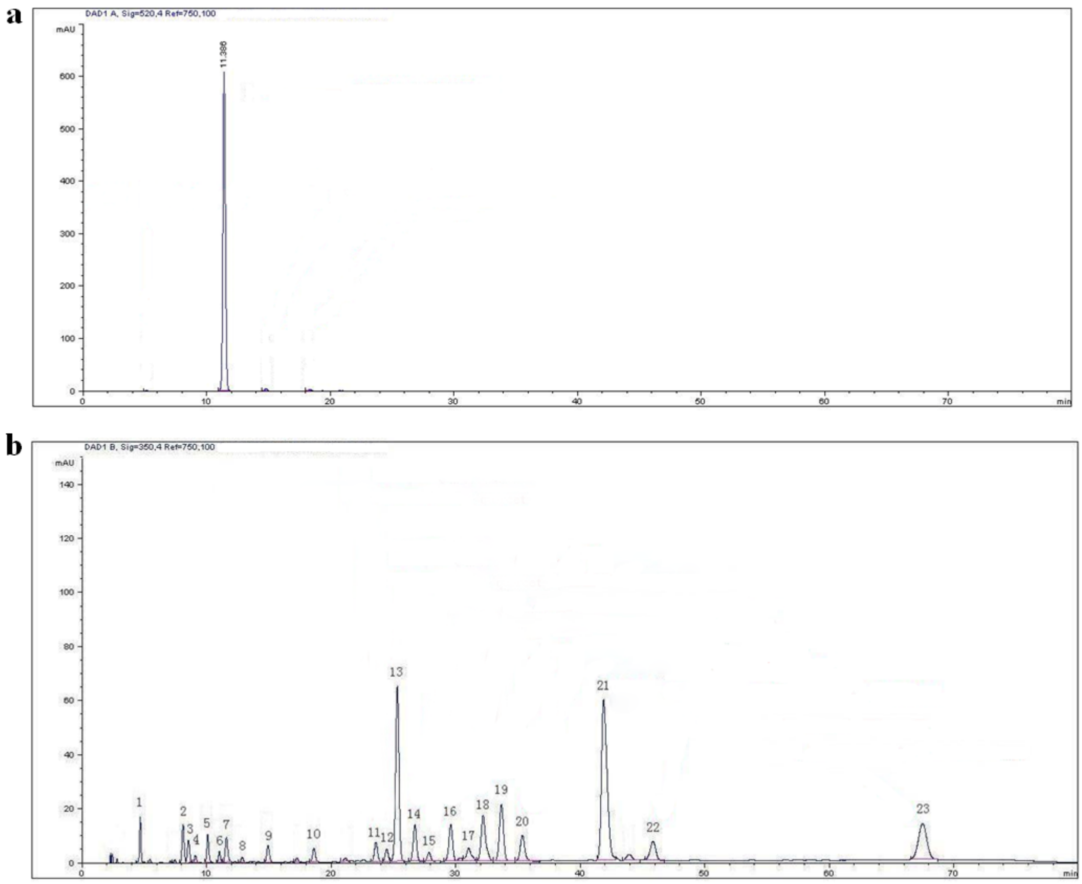

Figure S1. Standards used for HPLC analysis.

a. Chromatograms of the anthocyanins.

b. Chromatograms of the other flavonoids. No. 11, 12, 13, and 23 represent flavones; No. 14, 15, 16, 18, 19, 20, and 21 represent flavonols; No. 22 represents flavanols.
