# Supplementary material for: Factor Analysis of MYB Gene Expression and Flavonoid Affecting Petal Color in Three Crabapple Cultivars
Source: Front Plant Sci. 2017 Feb 7;8:137. doi: 10.3389/fpls.2017.00137 (PMC5293739; doi:10.3389/fpls.2017.00137)
Supplement: Supplementary file 9 [file Image2.PDF]

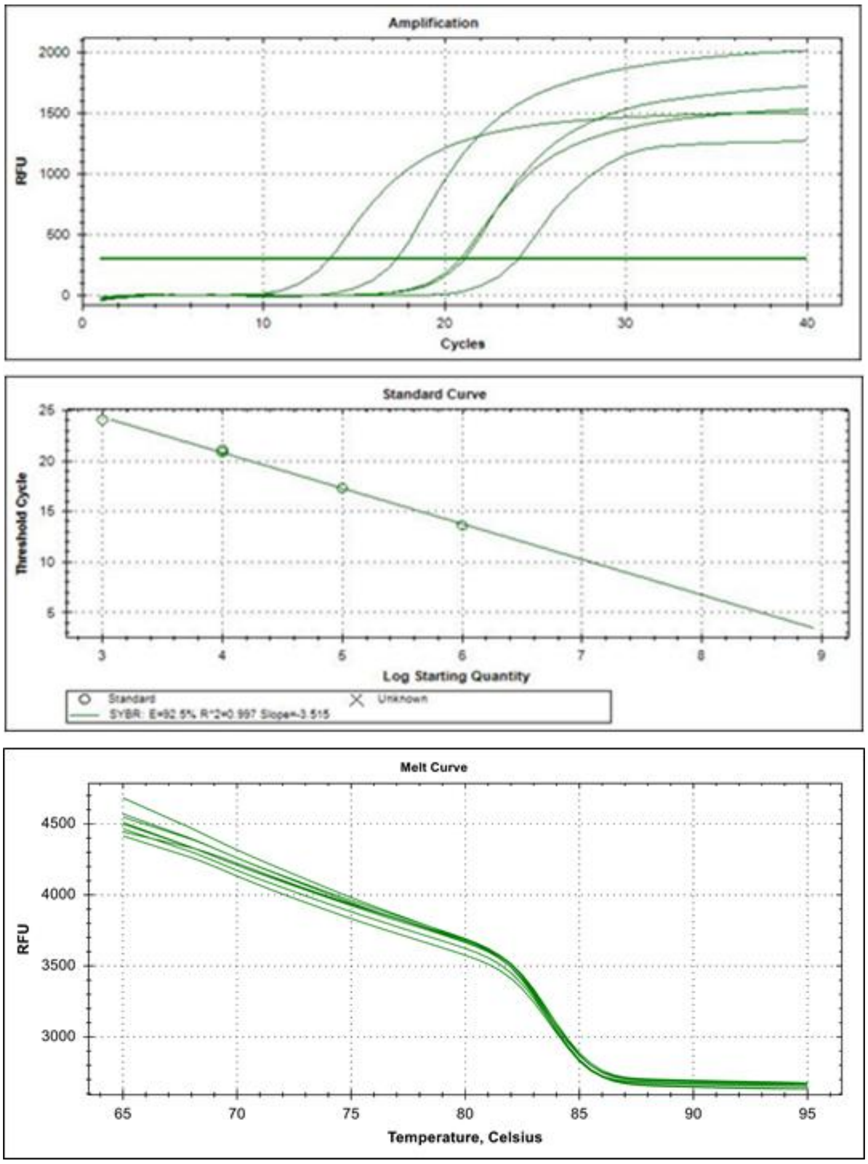

Fig S2-1 The Amplification, Standard Curve and Melt Curve of the primes of 18S

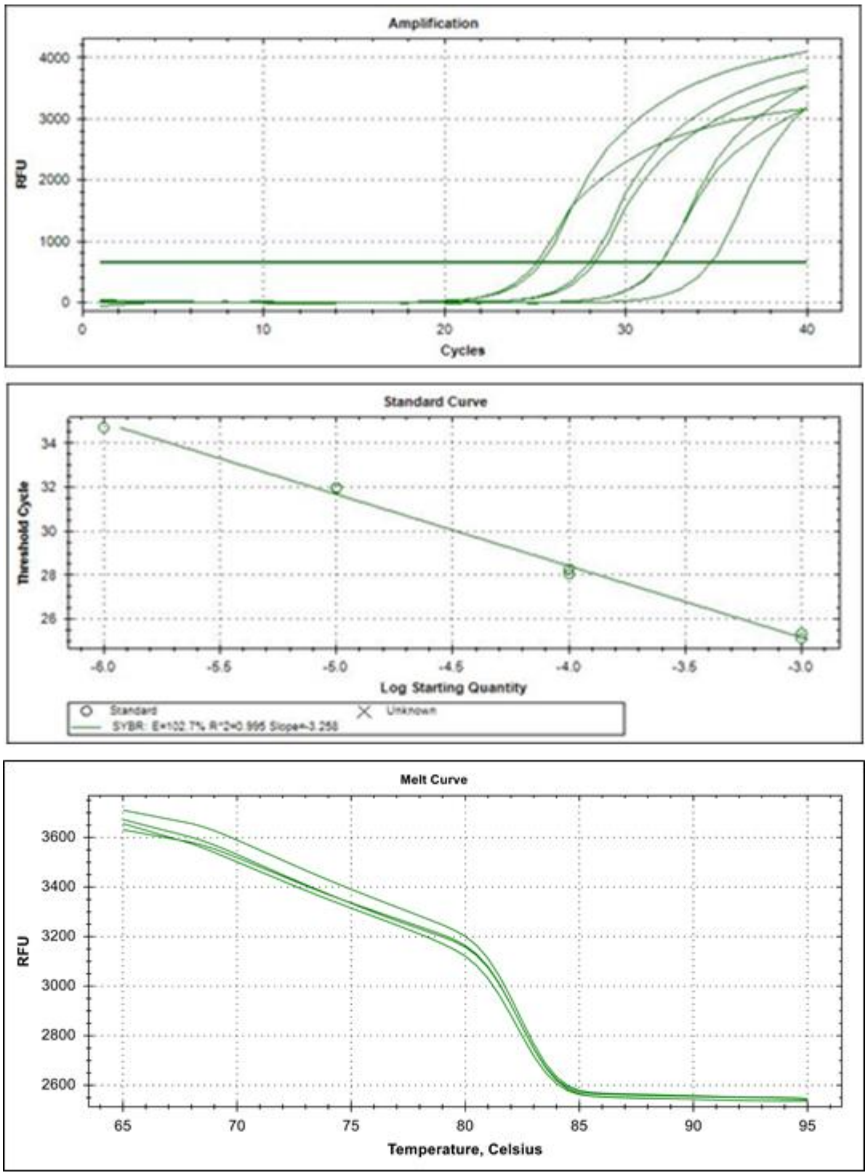

Fig S2-2 The Amplification, Standard Curve and Melt Curve of the primes of McPAL

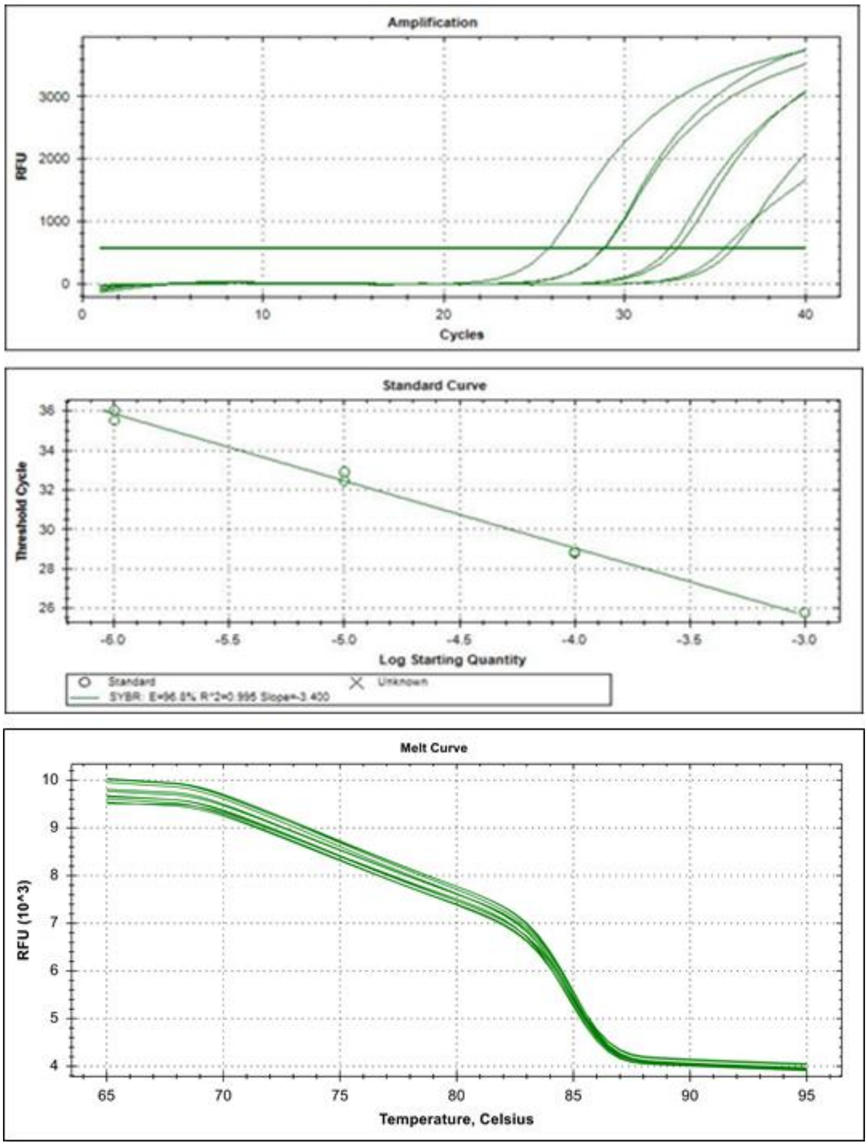

Fig S2-3 The Amplification and Standard Curve of the primes of McCHS

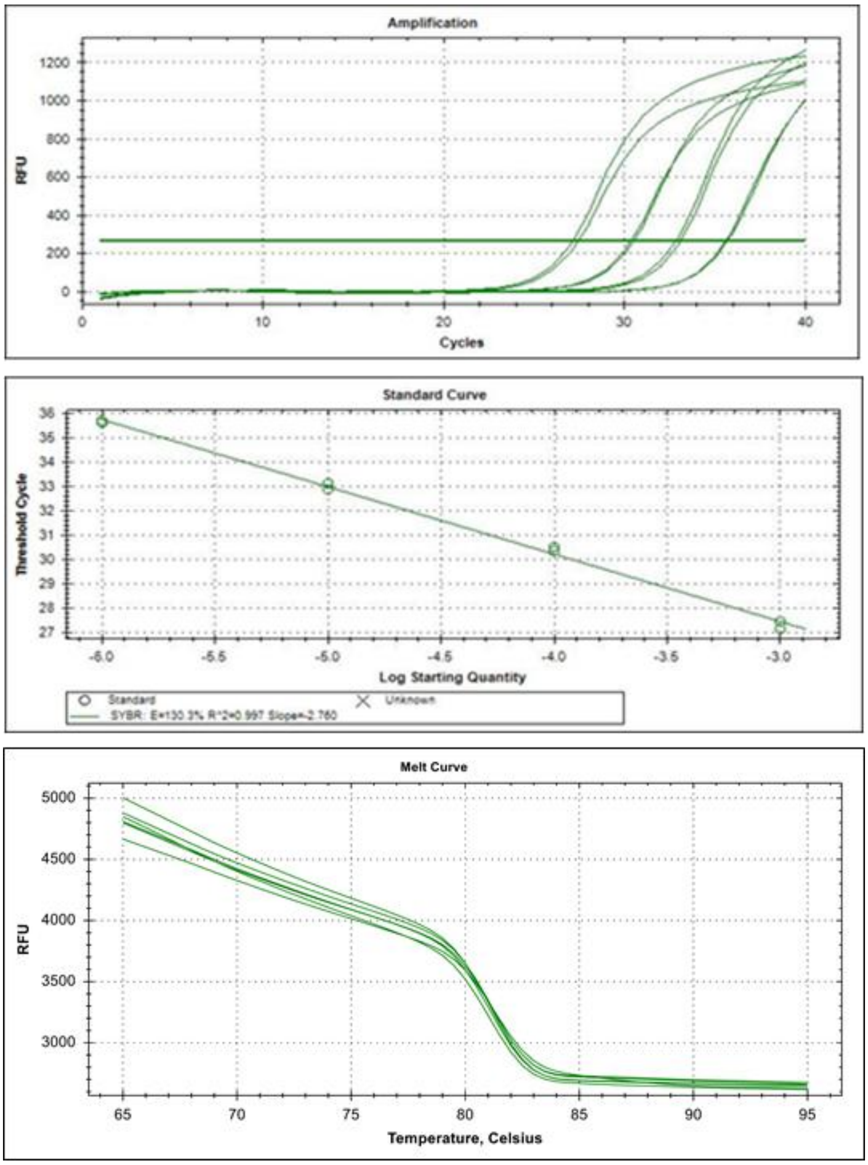

Fig S2-4 The Amplification, Standard Curve and Melt Curve of the primes of McCHI

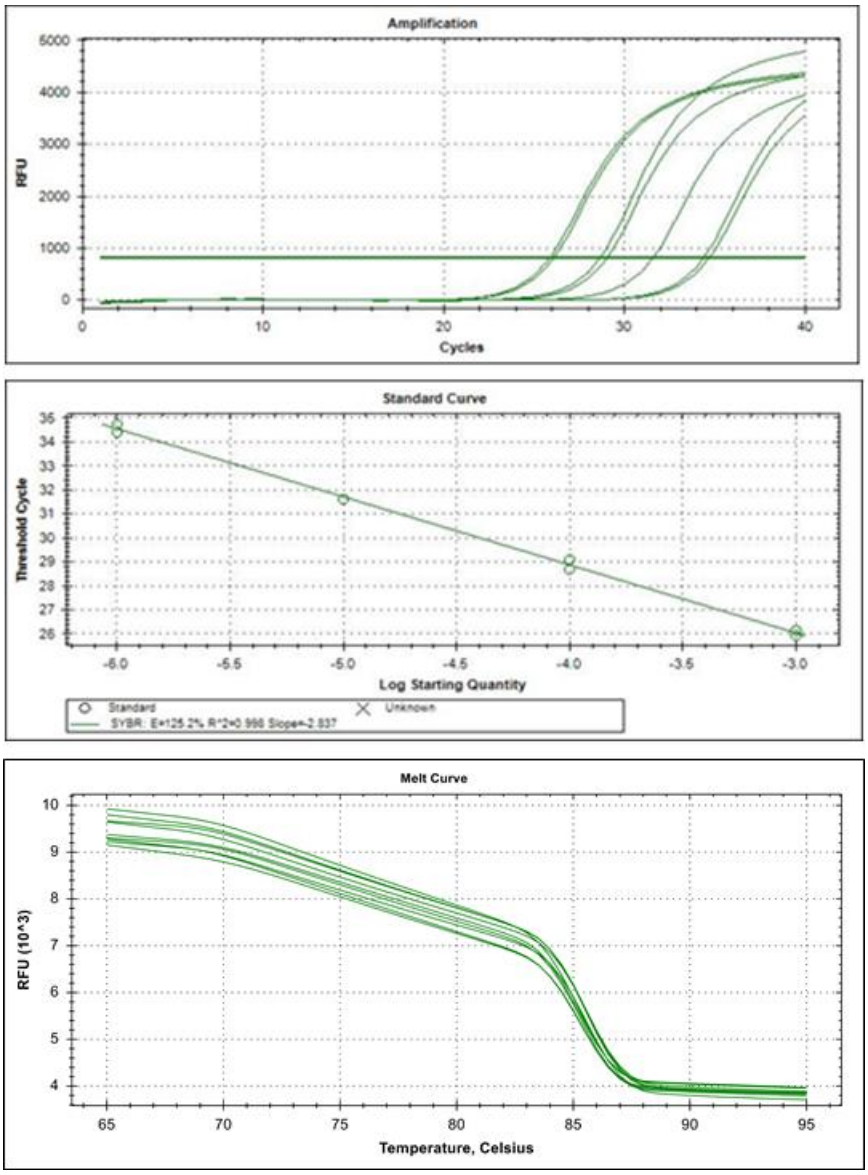

Fig S2-5 The Amplification, Standard Curve and Melt Curve of the primes of McF3H

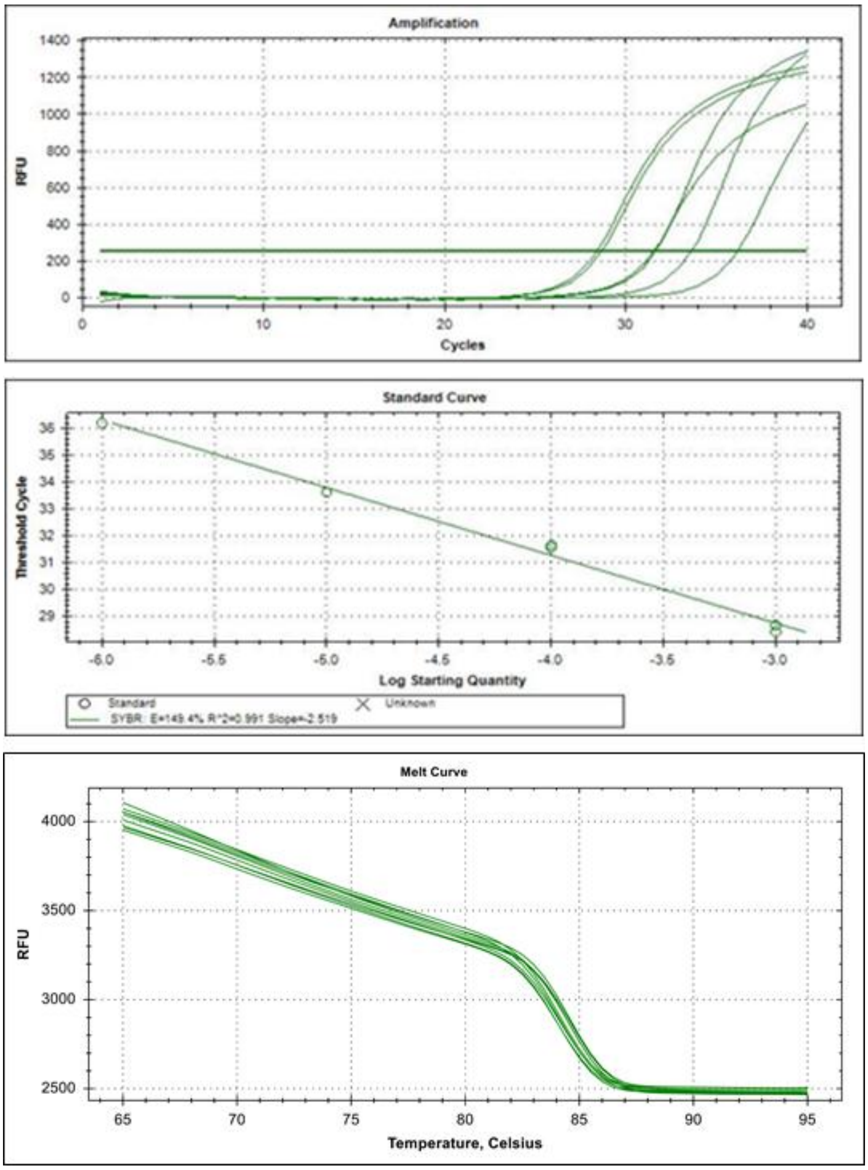

Fig S2-6 The Amplification, Standard Curve and Melt Curve of the primes of McF3’H

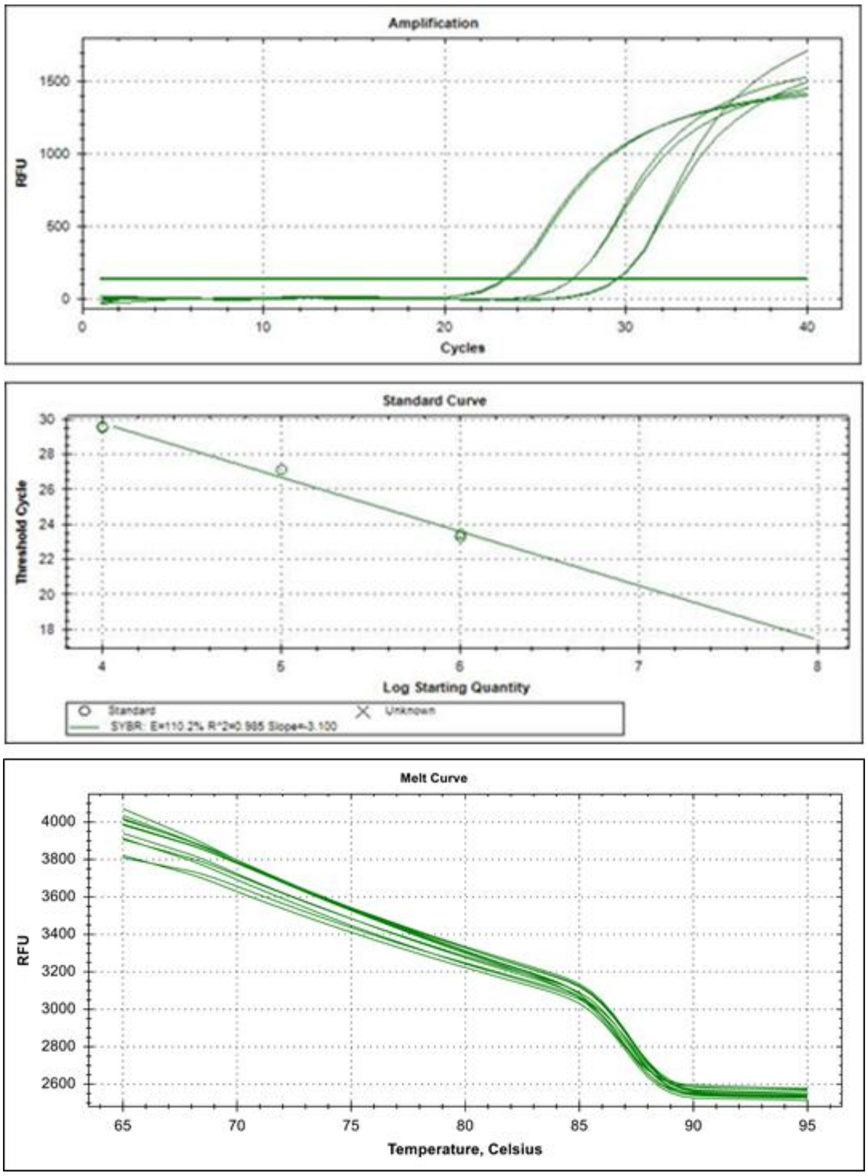

Fig S2-7 The Amplification, Standard Curve and Melt Curve of the primes of McDFR

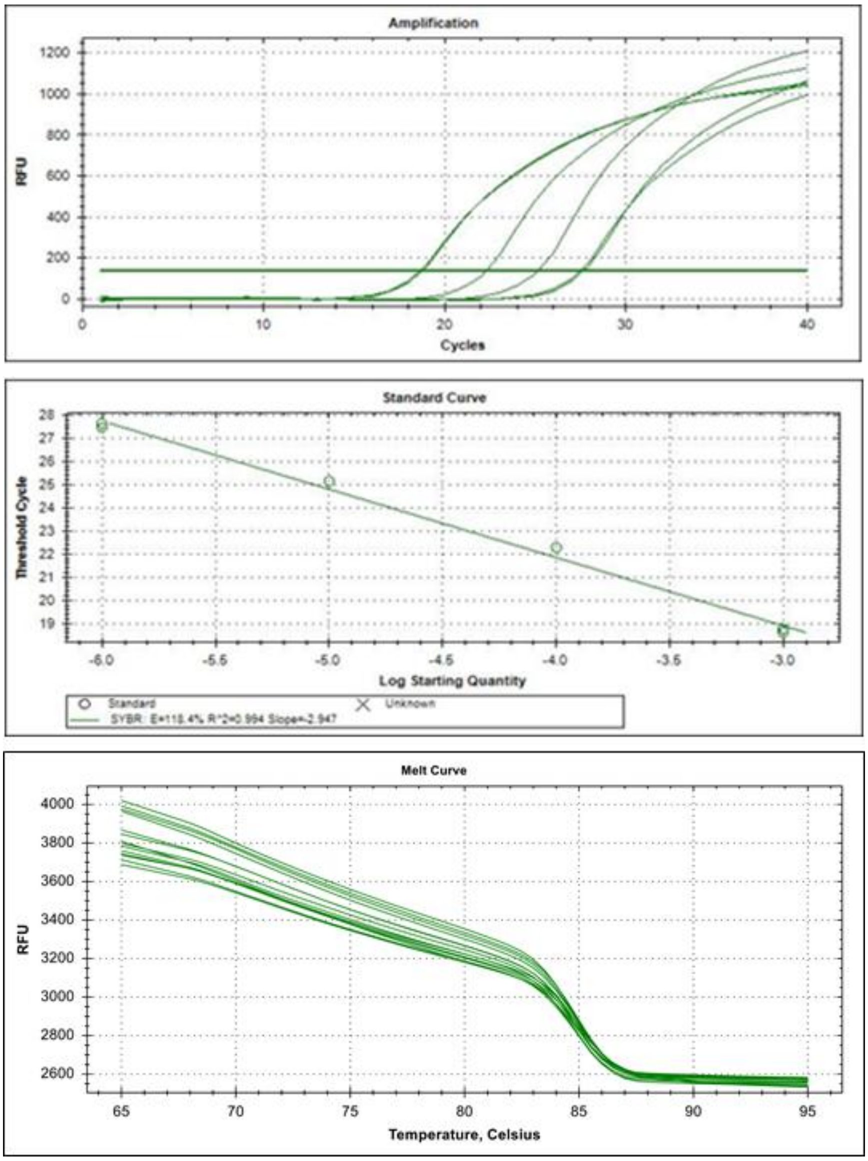

Fig S2-8 The Amplification, Standard Curve and Melt Curve of the primes of McANS

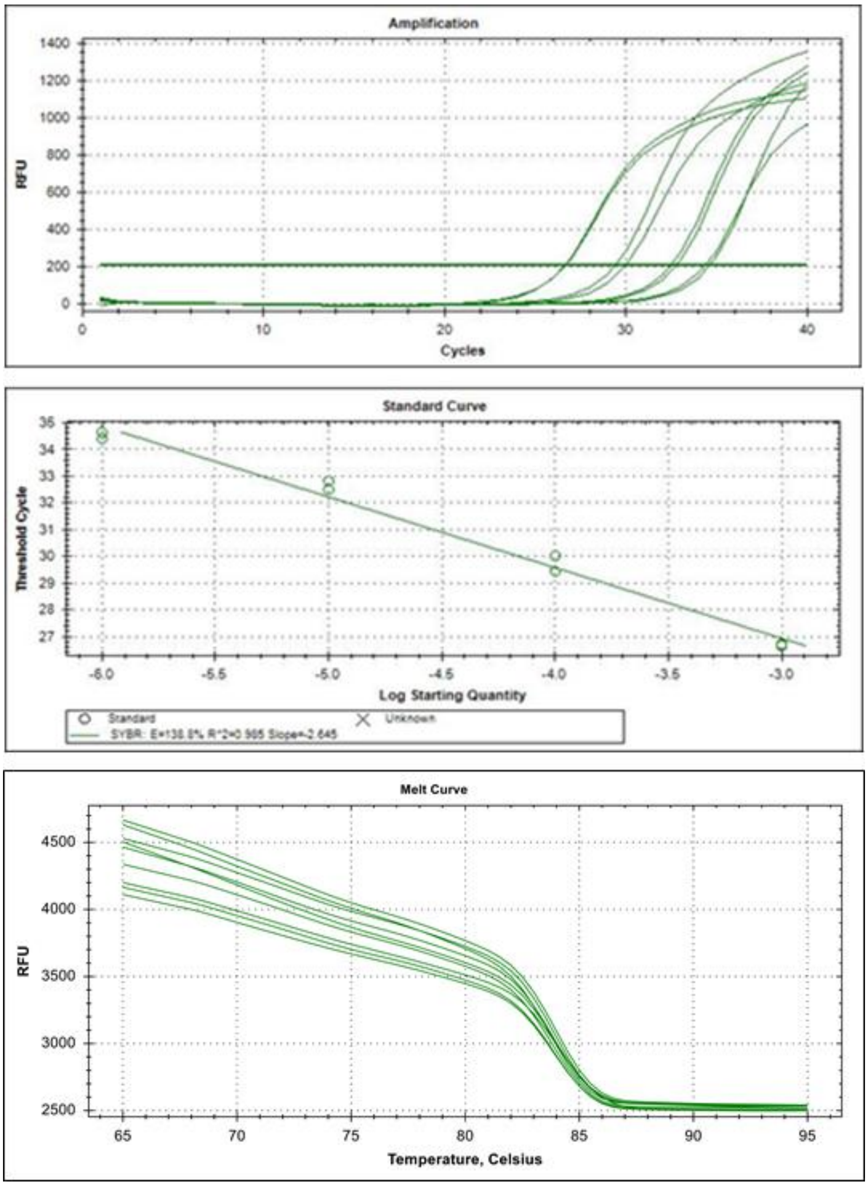

Fig S2-9 The Amplification, Standard Curve and Melt Curve of the primes of McUFGT

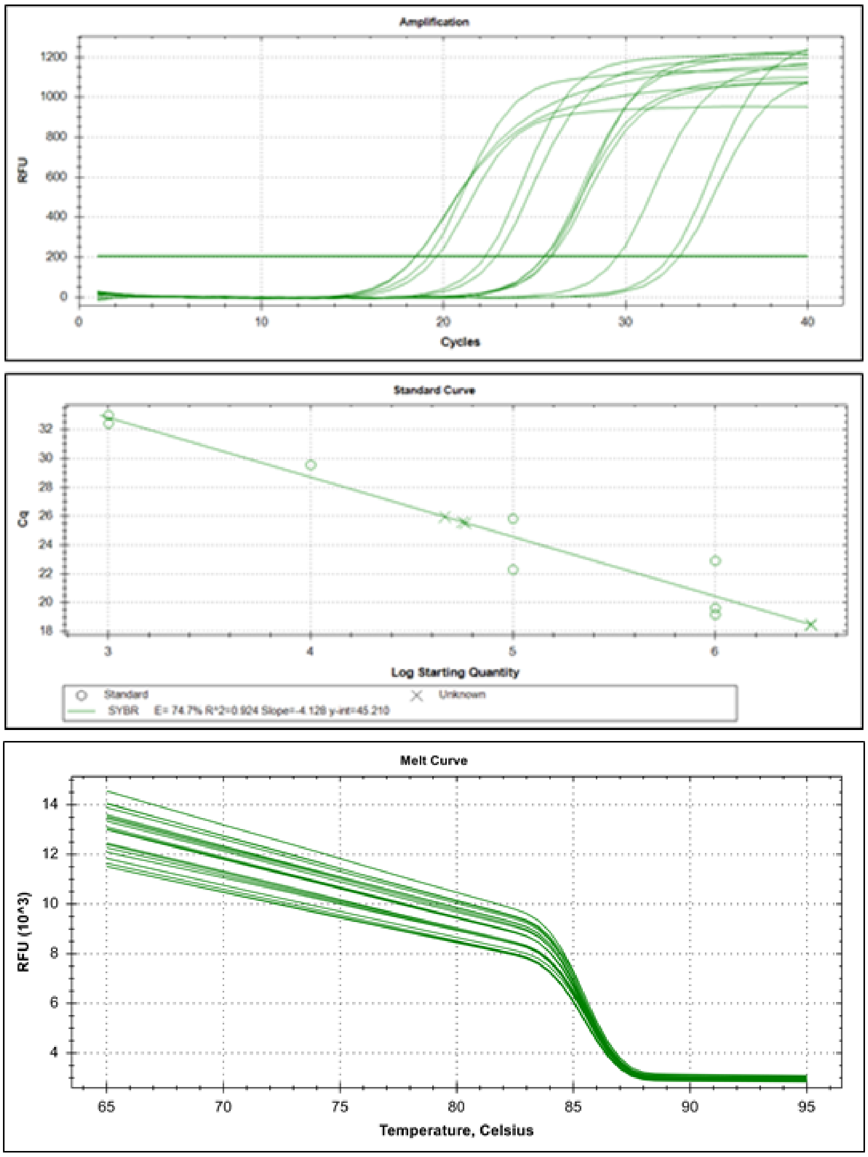

Fig S2-10 The Amplification, Standard Curve and Melt Curve of the primes of McLAR

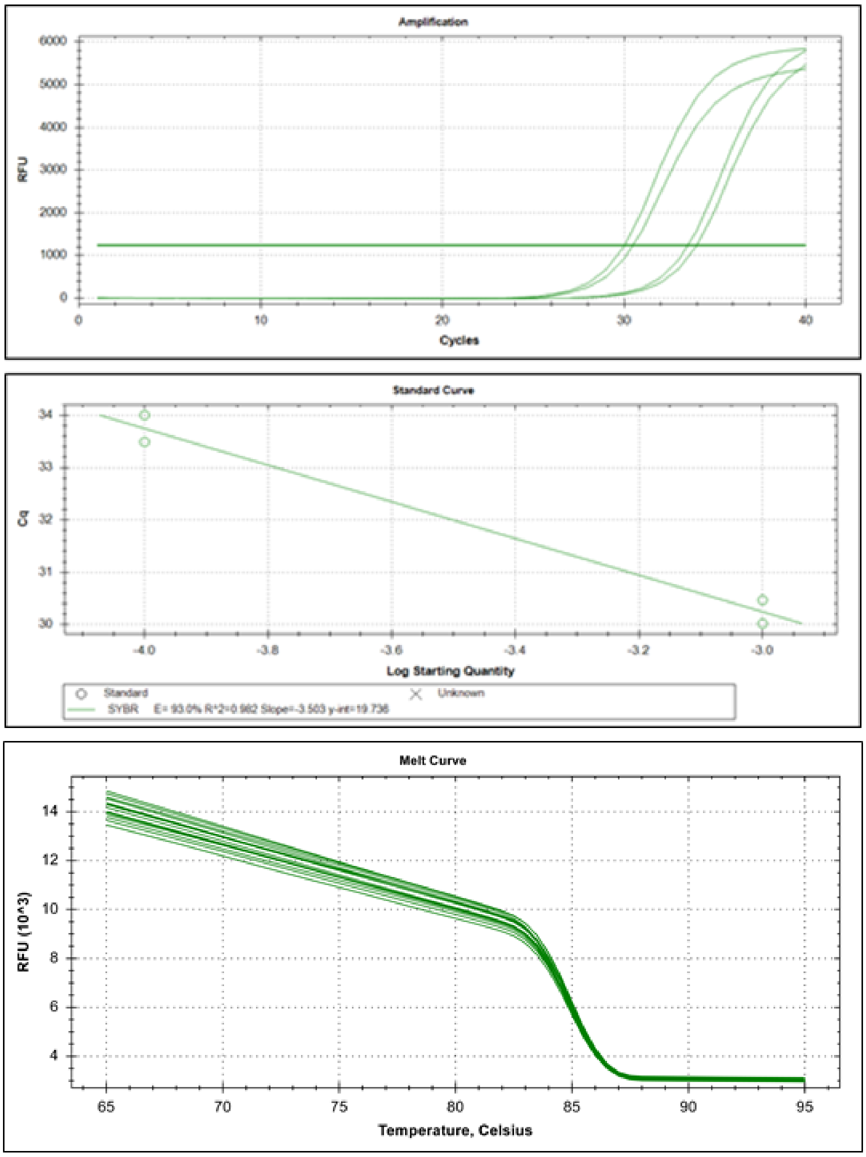

Fig S2-11 The Amplification, Standard Curve and Melt Curve of the primes of McANR

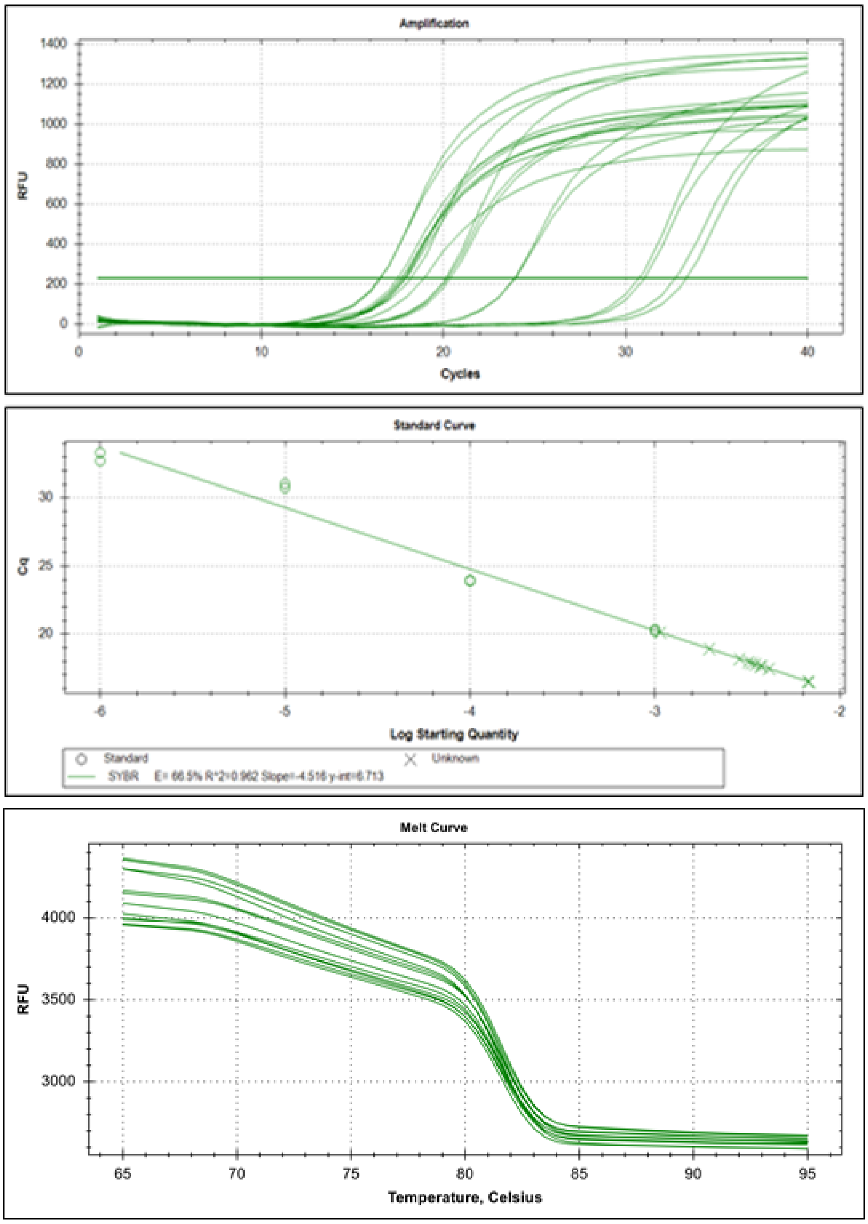

Fig S2-12 The Amplification, Standard Curve and Melt Curve of the primes of McFLS

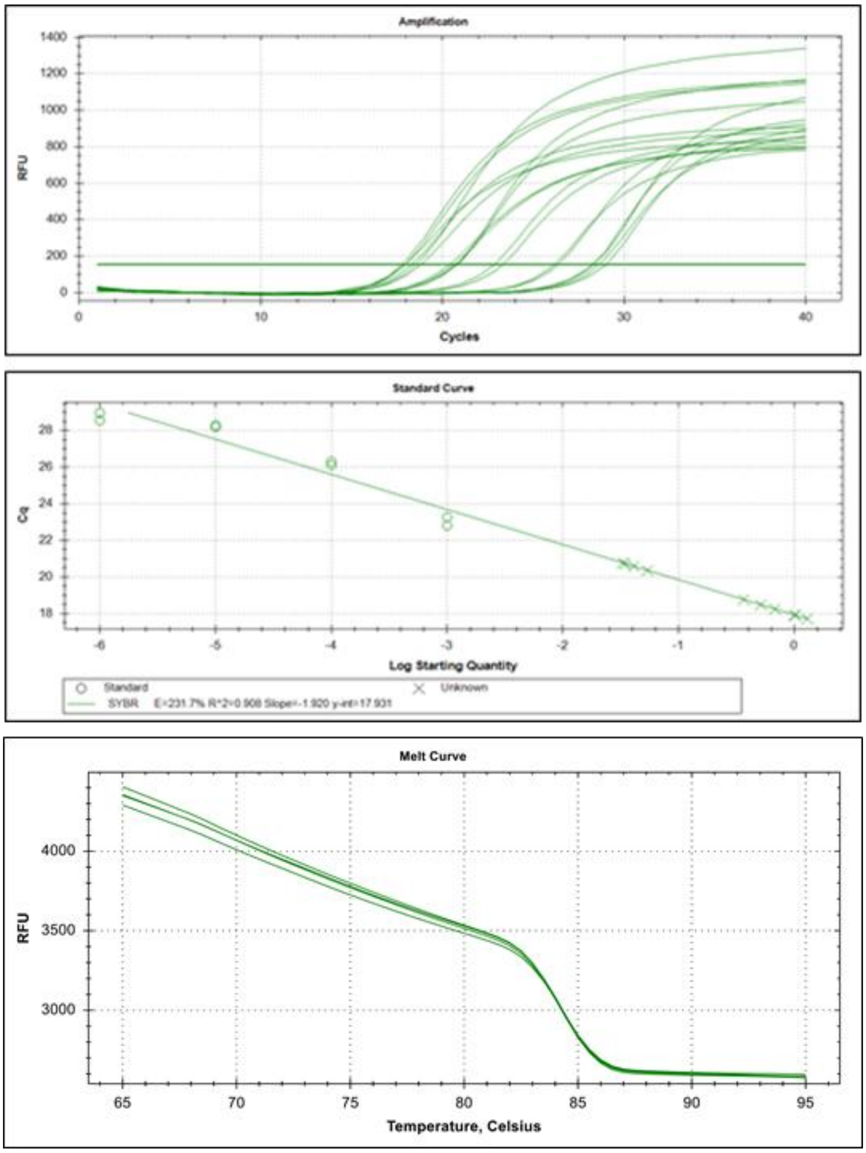

Fig S2-13 The Amplification, Standard Curve and Melt Curve of the primes of McMYB1

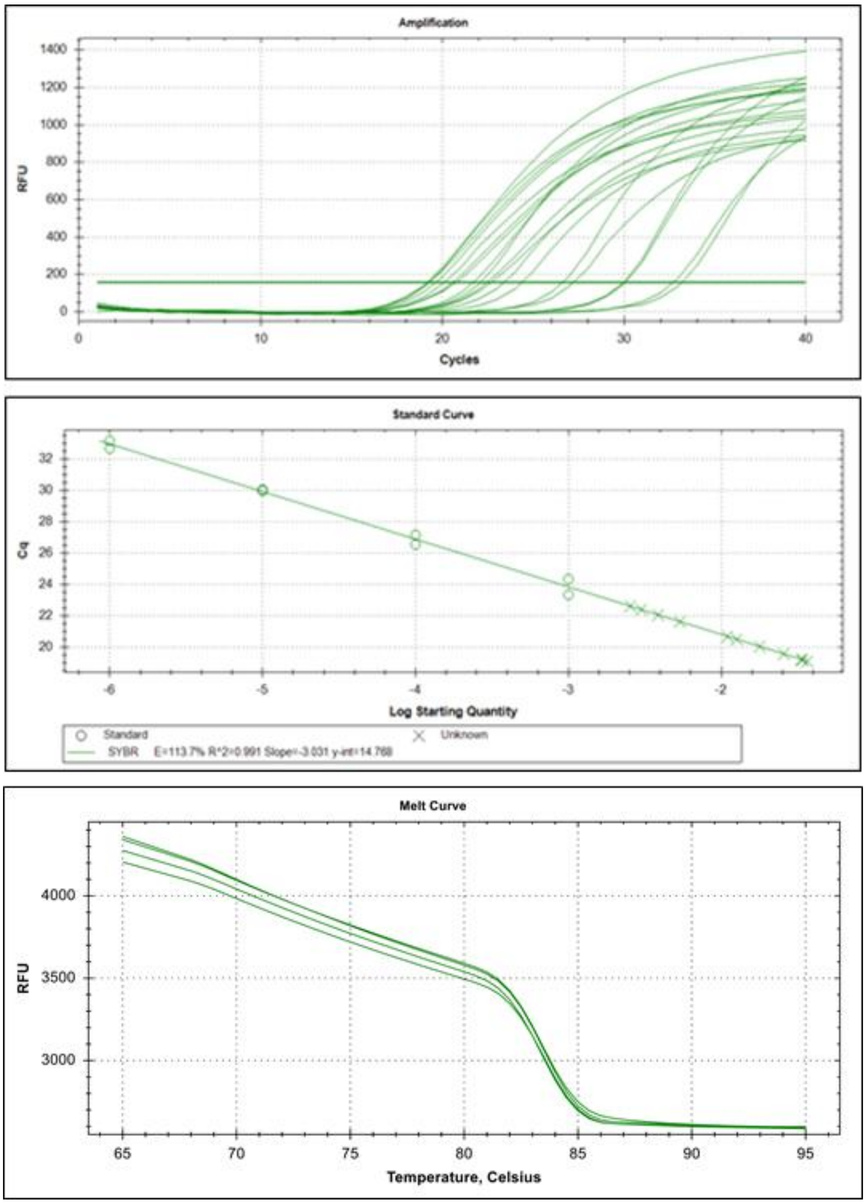

Fig S2-14 The Amplification, Standard Curve and Melt Curve of the primes of McMYB2

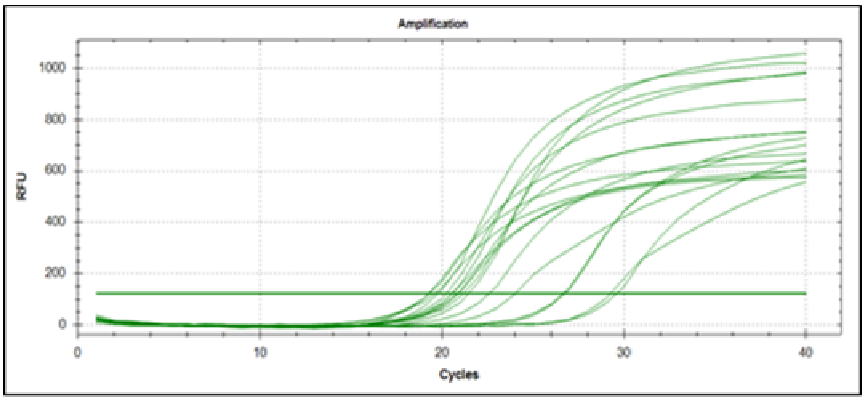

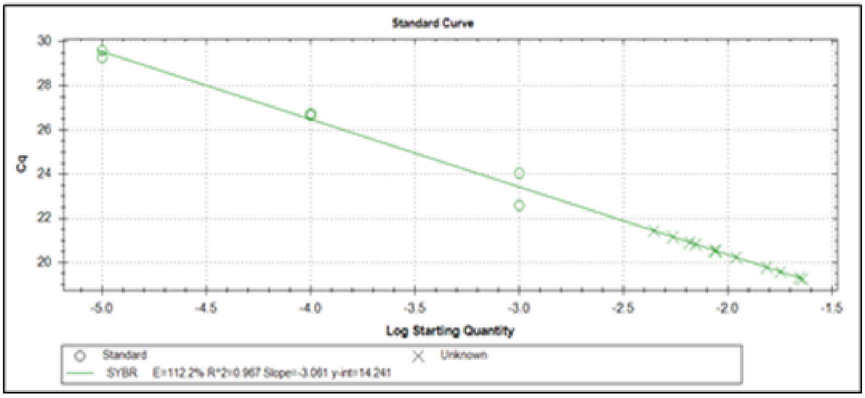

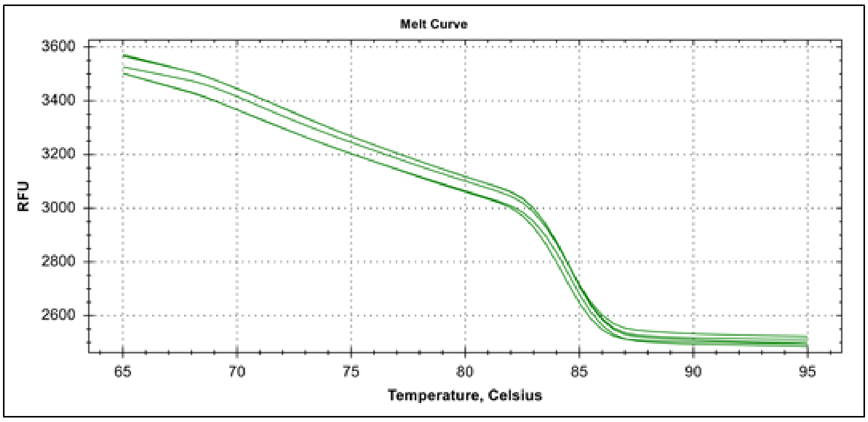

Fig S2-15 The Amplification, Standard Curve and Melt Curve of the primes of McMYB3

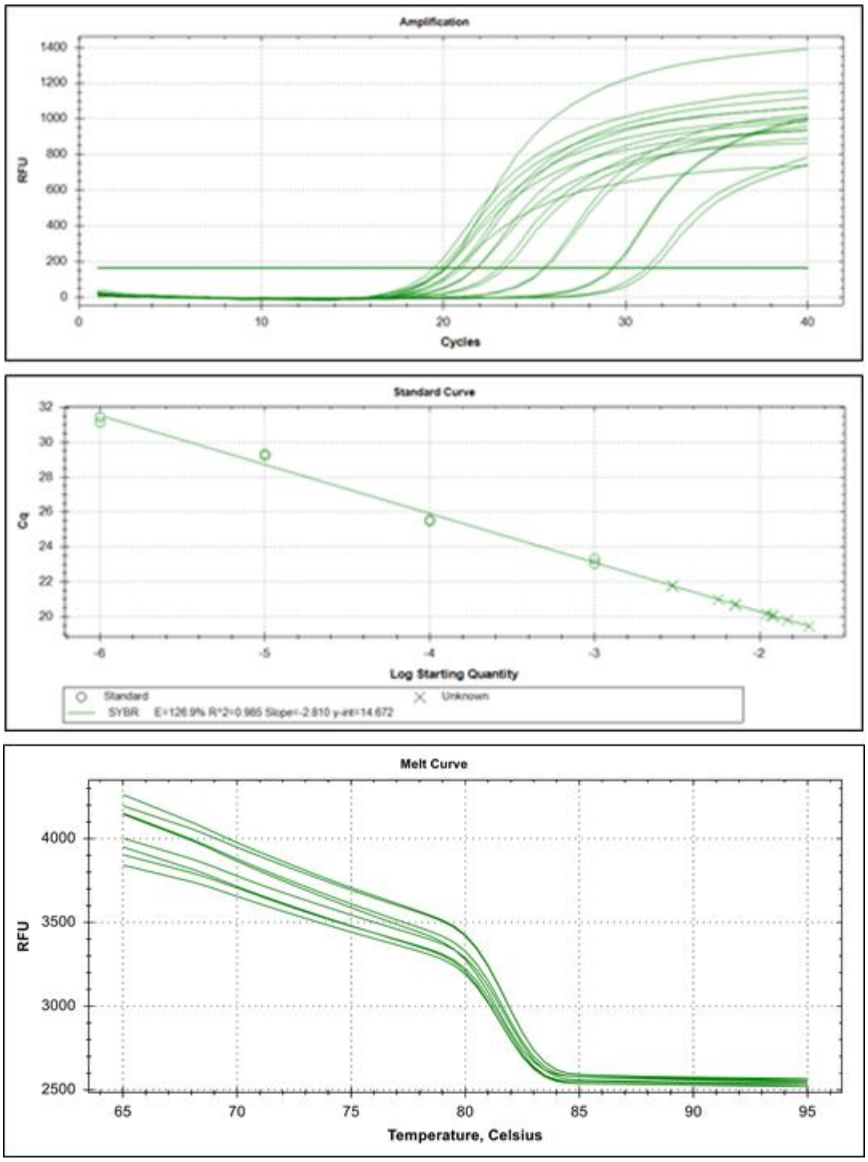

Fig S2-16 The Amplification, Standard Curve and Melt Curve of the primes of McMYB4

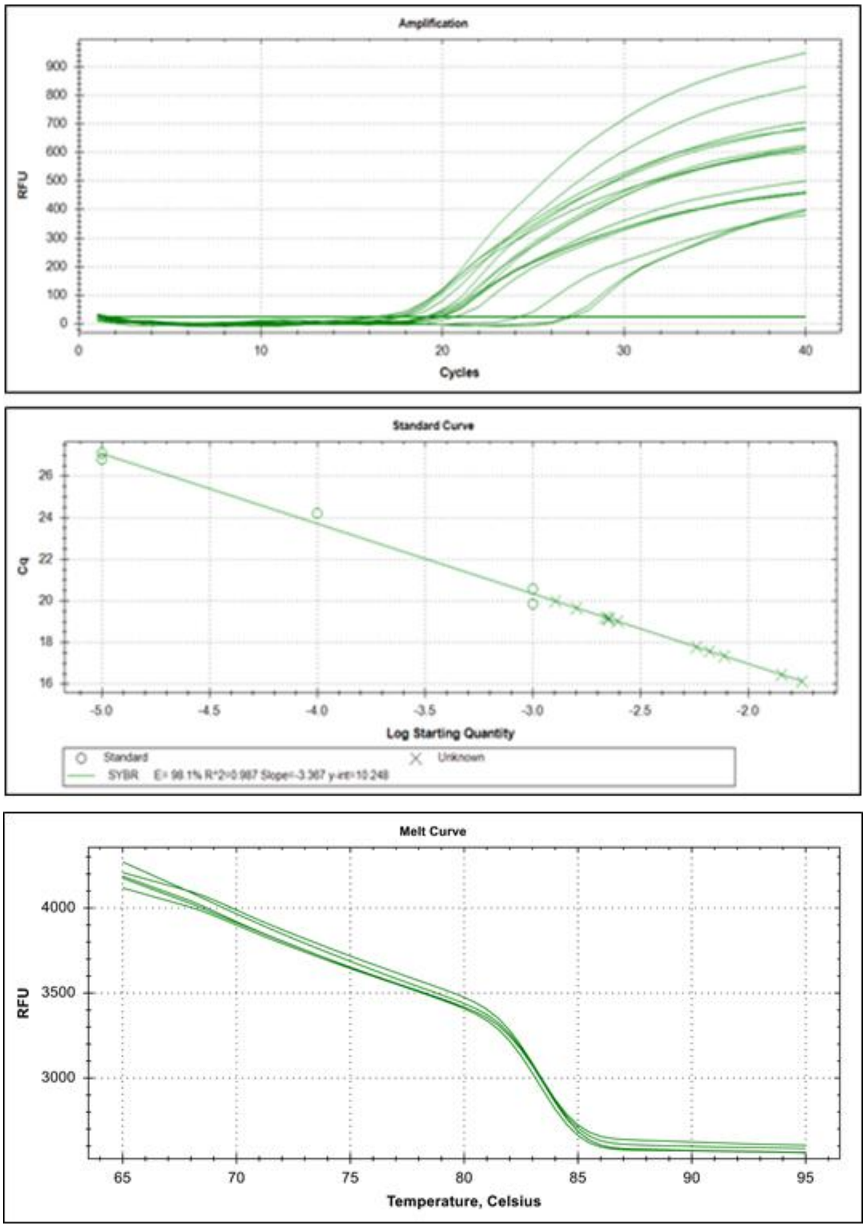

Fig S2-17 The Amplification, Standard Curve and Melt Curve of the primes of McMYB5

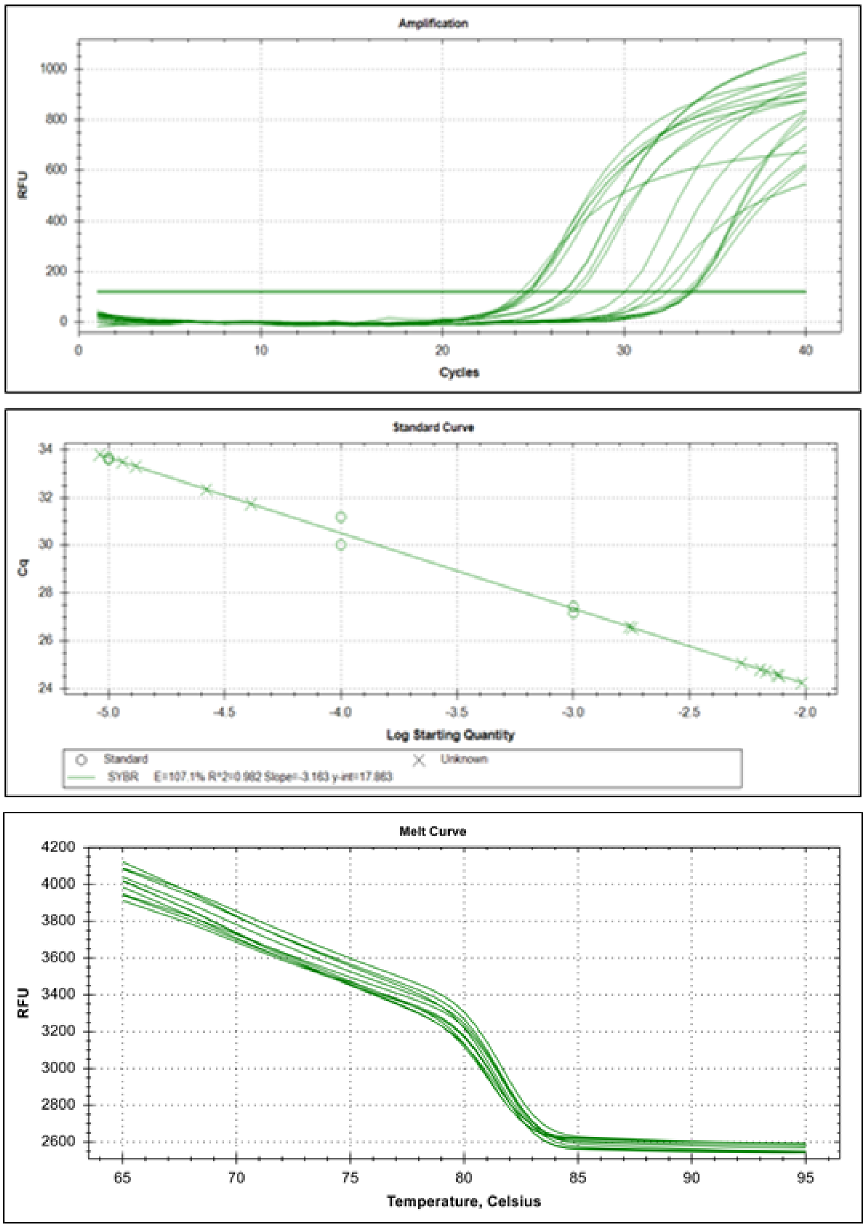

Fig S2-18 The Amplification, Standard Curve and Melt Curve of the primes of McMYB6

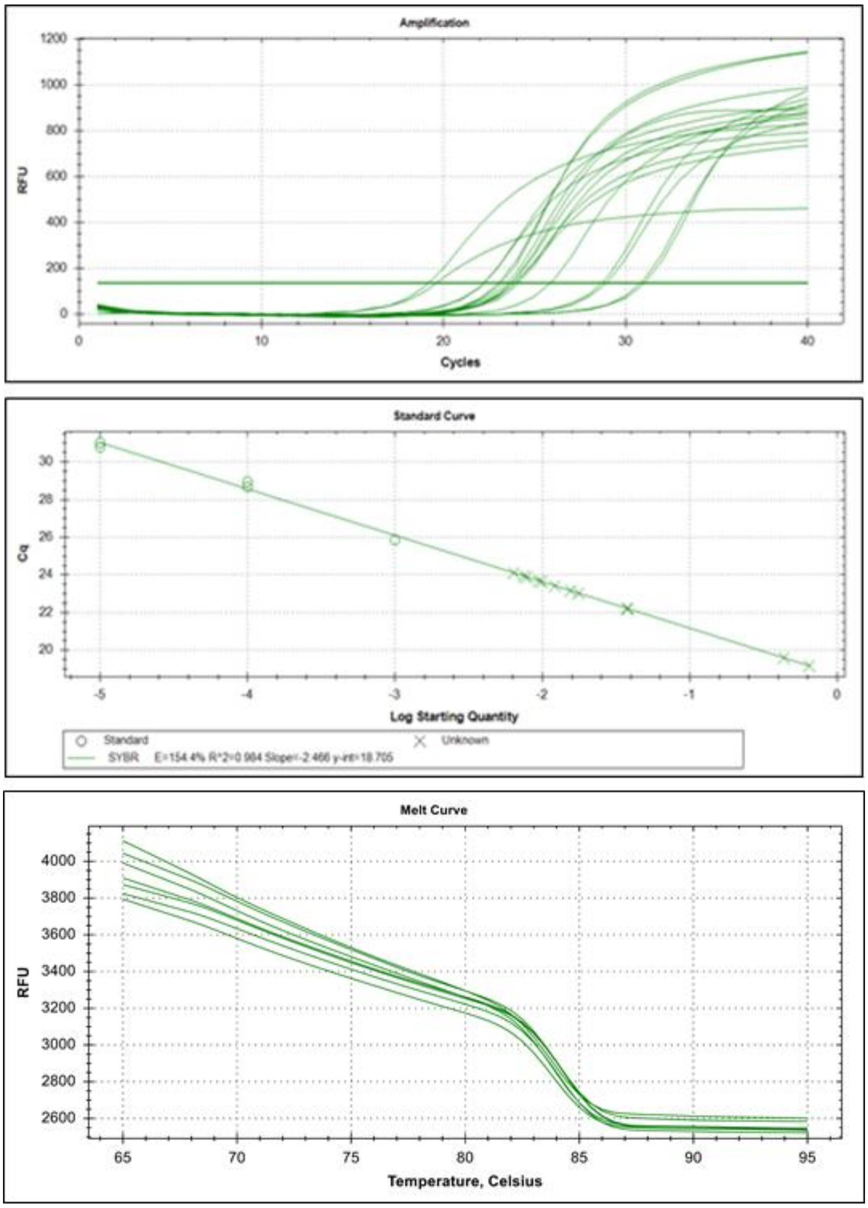

Fig S2-19 The Amplification, Standard Curve and Melt Curve of the primes of McMYB7

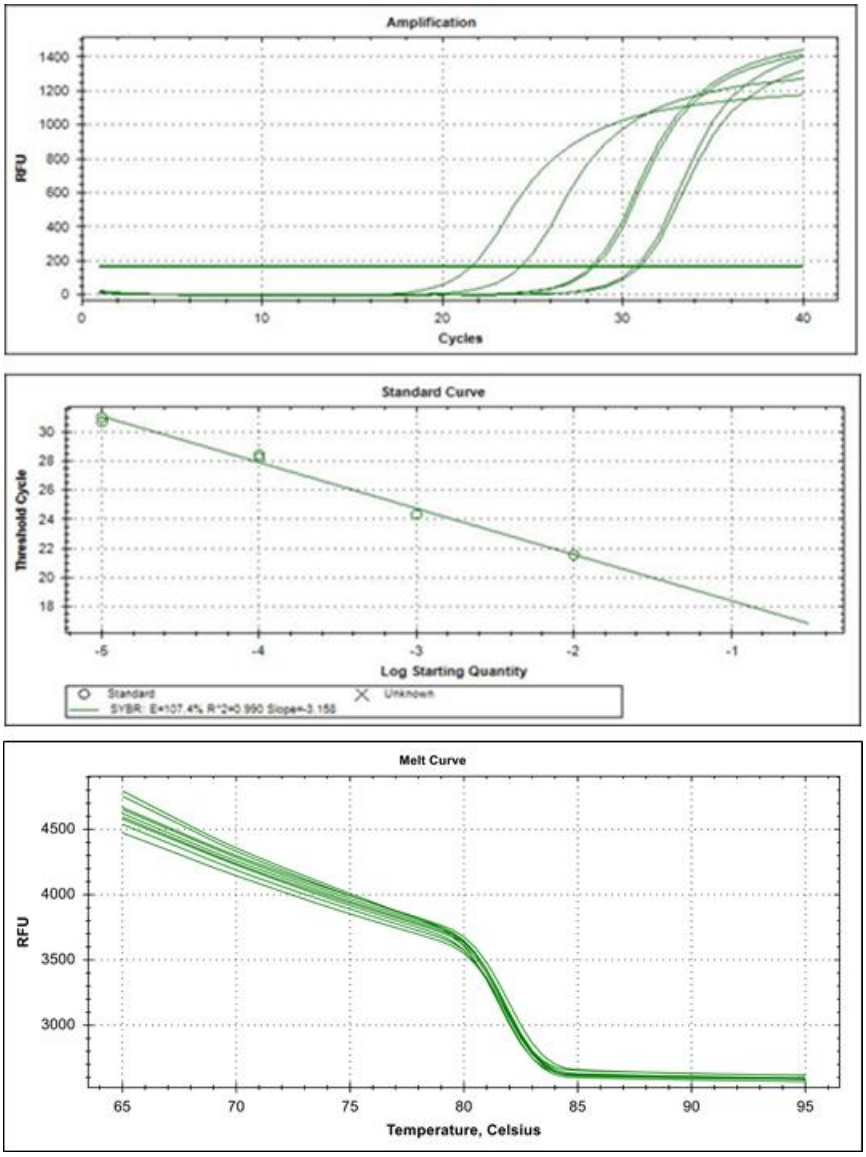

Fig S2-20 The Amplification, Standard Curve and Melt Curve of the primes of McMYB10

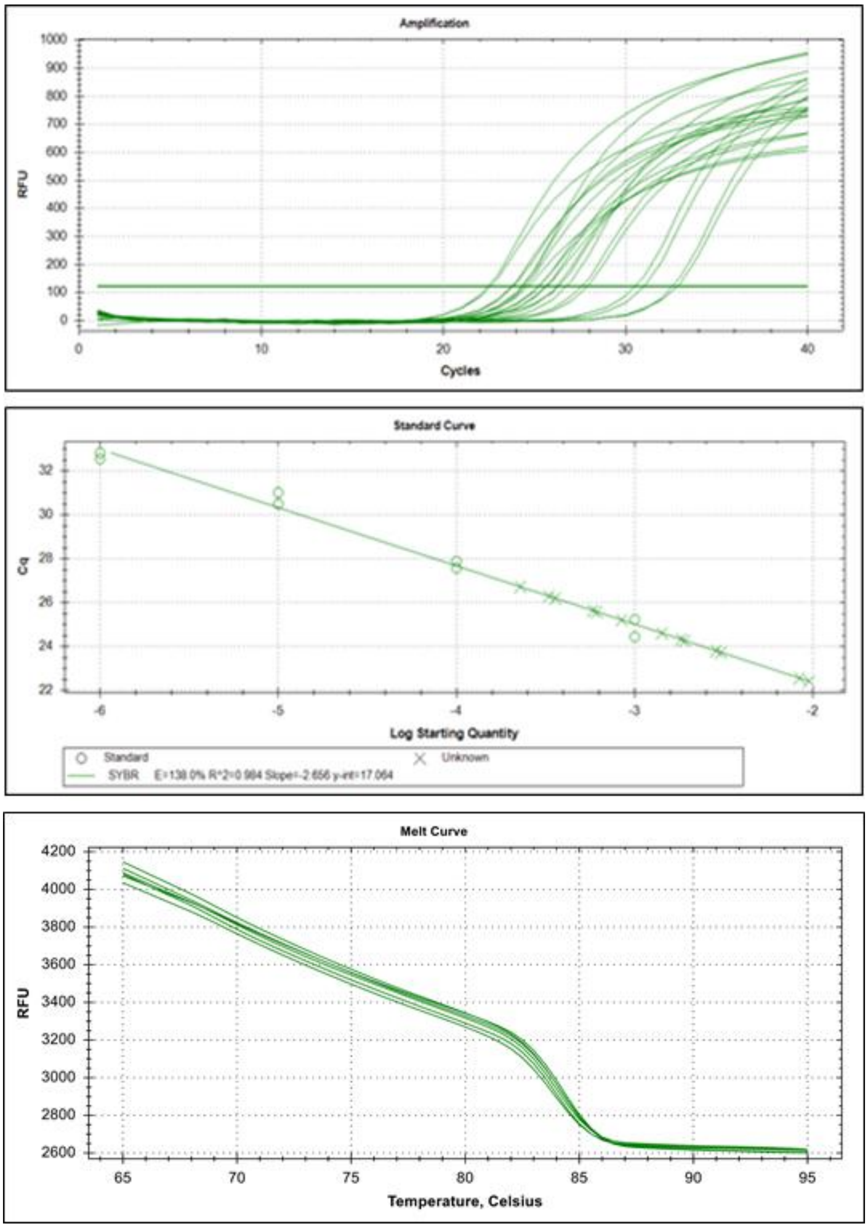

Fig S2-21 The Amplification, Standard Curve and Melt Curve of the primes of McMYB14
