# Supplementary material for: Factor Analysis of MYB Gene Expression and Flavonoid Affecting Petal Color in Three Crabapple Cultivars
Source: Front Plant Sci. 2017 Feb 7;8:137. doi: 10.3389/fpls.2017.00137 (PMC5293739; doi:10.3389/fpls.2017.00137)
Supplement: Supplementary file 10 [file Image3.PDF]

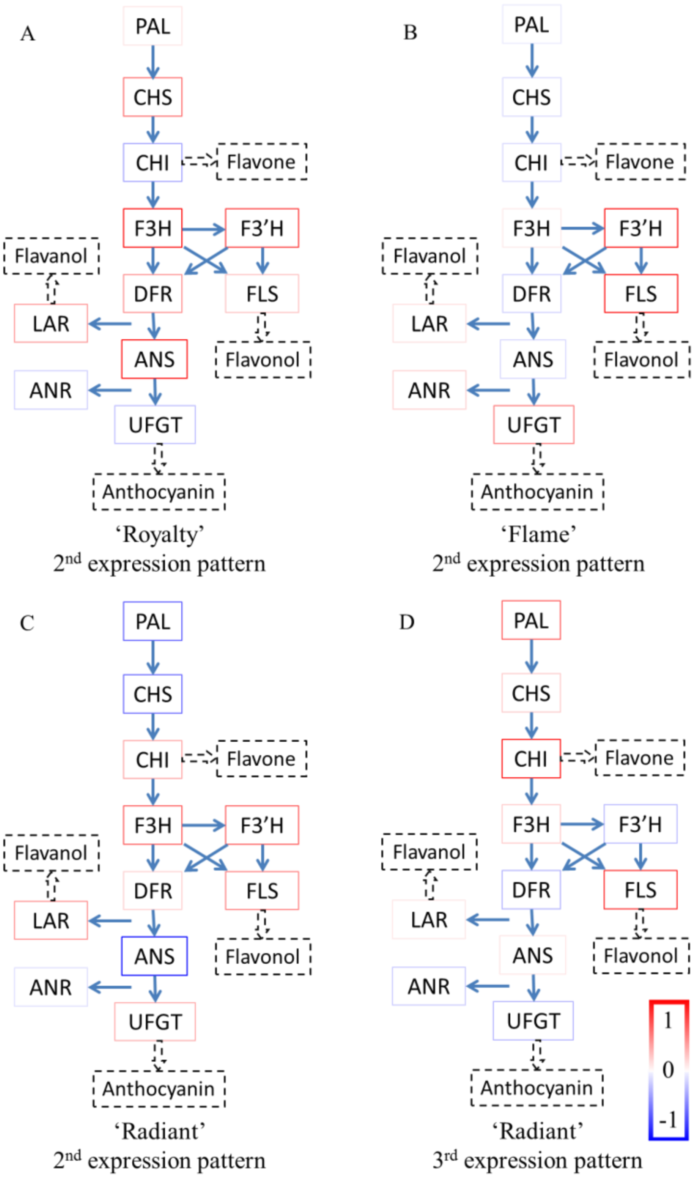

Figure S3. The other results from the factor analysis of the expression levels of the structure genes. The results calculated between -1 and 1 are expressed as boxes in the colors shown at the bottom right. The boxes for each gene were integrated into one pathway for each of the three cultivars.
